# Topological Small-World Organization of the Fibroblastic Reticular Cell Network Determines Lymph Node Functionality

**DOI:** 10.1371/journal.pbio.1002515

**Published:** 2016-07-14

**Authors:** Mario Novkovic, Lucas Onder, Jovana Cupovic, Jun Abe, David Bomze, Viviana Cremasco, Elke Scandella, Jens V. Stein, Gennady Bocharov, Shannon J. Turley, Burkhard Ludewig

**Affiliations:** 1 Institute of Immunobiology, Kantonsspital St. Gallen, St. Gallen, Switzerland; 2 Theodor Kocher Institute, University of Bern, Bern, Switzerland; 3 Novartis Institutes for Biomedical Research, Cambridge, Massachusetts, United States of America; 4 Institute of Numerical Mathematics, Russian Academy of Sciences, Moscow, Russia; 5 Department of Cancer Immunology, Genentech, South San Francisco, California, United States of America; ETH Zurich, SWITZERLAND

## Abstract

Fibroblastic reticular cells (FRCs) form the cellular scaffold of lymph nodes (LNs) and establish distinct microenvironmental niches to provide key molecules that drive innate and adaptive immune responses and control immune regulatory processes. Here, we have used a graph theory-based systems biology approach to determine topological properties and robustness of the LN FRC network in mice. We found that the FRC network exhibits an imprinted small-world topology that is fully regenerated within 4 wk after complete FRC ablation. Moreover, in silico perturbation analysis and in vivo validation revealed that LNs can tolerate a loss of approximately 50% of their FRCs without substantial impairment of immune cell recruitment, intranodal T cell migration, and dendritic cell-mediated activation of antiviral CD8^+^ T cells. Overall, our study reveals the high topological robustness of the FRC network and the critical role of the network integrity for the activation of adaptive immune responses.

## Abstract 2

Topological complex network analysis reveals an underlying robust "small-world" organization of fibroblastic reticular cells and its critical role in the maintenance of lymph node functionality.

## Author Summary

Fibroblastic reticular cells (FRCs) in lymph nodes are organized in a highly connected cellular network that not only acts as a scaffold for lymphocyte migration but also provides key factors for induction and maintenance of immune responses. By utilizing high-resolution microscopy coupled with computational approaches to complex network analysis, we determined the topological properties and robustness of the FRC network. The underlying structure of the FRC network has been identified as a small-world network analogous to many other biological networks. Moreover, we demonstrate that this distinct structural organization is an imprinted trait of the FRC network, which is capable of fully regenerating after complete FRC ablation. In silico perturbation analysis of the FRC network confirmed that lymph nodes are able to tolerate FRC loss of approximately 50%. In vivo experiments corroborated these findings by demonstrating substantial impairment of immune cell recruitment, migration, and dendritic cell-mediated activation of antiviral CD8^+^ T cells, after critical loss of FRCs. In conclusion, the present study reveals the extraordinary topological robustness of the FRC network, crucial for establishing effective immunity.

## Introduction

Efficient interactions between the immune system and microbial antigens are initiated and maintained in secondary lymphoid organs (SLOs) that are strategically positioned at routes of pathogen invasion. Lymph nodes (LNs), for example, are found at convergence points of larger lymph vessels, which drain extracellular fluids from peripheral tissues [1]. The interaction of naïve T cells with antigen-presenting dendritic cells (DCs) in LNs needs to be well coordinated because T cells with a particular specificity are rare [2,3]. Optimal communication between immune cells relies to a large extent on the fibroblastic reticular cell (FRC) network that provides specialized microenvironments for cellular interactions. For example, FRCs regulate T cell migration and survival in the T cell zone by producing homeostatic chemokines and cytokines [4–6]. Moreover, FRCs located in and around B cell follicles coordinate B cell trafficking and activity [7–9]. Importantly, while the role of FRCs in the regulation of immune responsiveness has been studied extensively (reviewed in [10,11]), the underlying principles of the FRC network topology and its contribution to general LN functionality have remained unexplored.

In order to determine the topological properties of networks, the theoretical framework of the graph theory can be utilized [12,13]. The theory of complex networks has been applied in the study of real-world networks, including the internet [14,15], scientific collaboration [16], power grid systems [17], and the worldwide air transportation network [18]. Moreover, graph theory has been instrumental for the analysis of various biological systems, such as metabolic networks [19,20], protein–protein interactions [21], and neuronal cell connectivity [22,23]. Different classes of networks can be defined based on the nature of their topology. Random networks are described by the Erdos-Renyi model [24] in which objects (nodes) form random connections (edges) between each other with the same probability. Hence, most nodes will have approximately the same number of connections, centered on the network average with a Poisson degree distribution. In contrast, scale-free networks [25,26] are characterized by a power-law degree distribution with most nodes possessing few connections and very few nodes showing large numbers of connections. These few highly connected nodes are called hubs, and they maintain the whole network structure. Networks with less-centralized structures are called small-world networks [27], where any two nodes can be reached with only a few steps in the network.

A key feature of complex networks is their robustness to perturbation, which denotes the ability of a network to remain operational when nodes are functionally impaired or destroyed [14]. Such topological robustness is determined by the organizational principles of the network and has an impact on overall network functionality [13]. Interestingly, most real-world networks exhibit small-world topology, a property that is thought to provide networks with high resilience to external perturbation [28]. In contrast to engineered systems, understanding biological robustness is a difficult challenge due to the multilayered complexity of the system in which functionally relevant measures of robustness need to be established [29].

The FRC network can be almost completely destroyed during viral infection [4] or substantially altered during chronic infection with parasites [30] leading to severe immune deficiency. It is therefore important to assess the topological robustness of the FRC network and to define those parameters that determine network resilience. To address these questions, we have utilized the *Ccl19*^*idtr*^ mouse model [7], which enables diphtheria toxin (DT)-mediated ablation of FRCs expressing C-C motif chemokine 19 (CCL19). Graph theory-based analysis showed that the LN FRC network forms a lattice-like small-world network that exhibits complex network topology, substantial connectivity, and high capacity for clustering. Moreover, in silico analysis and thorough in vivo validation revealed substantial topological robustness of the FRC network.

## Results

### Topological Small-World Organization of the T Cell Zone FRC Network

**Fig 1:**
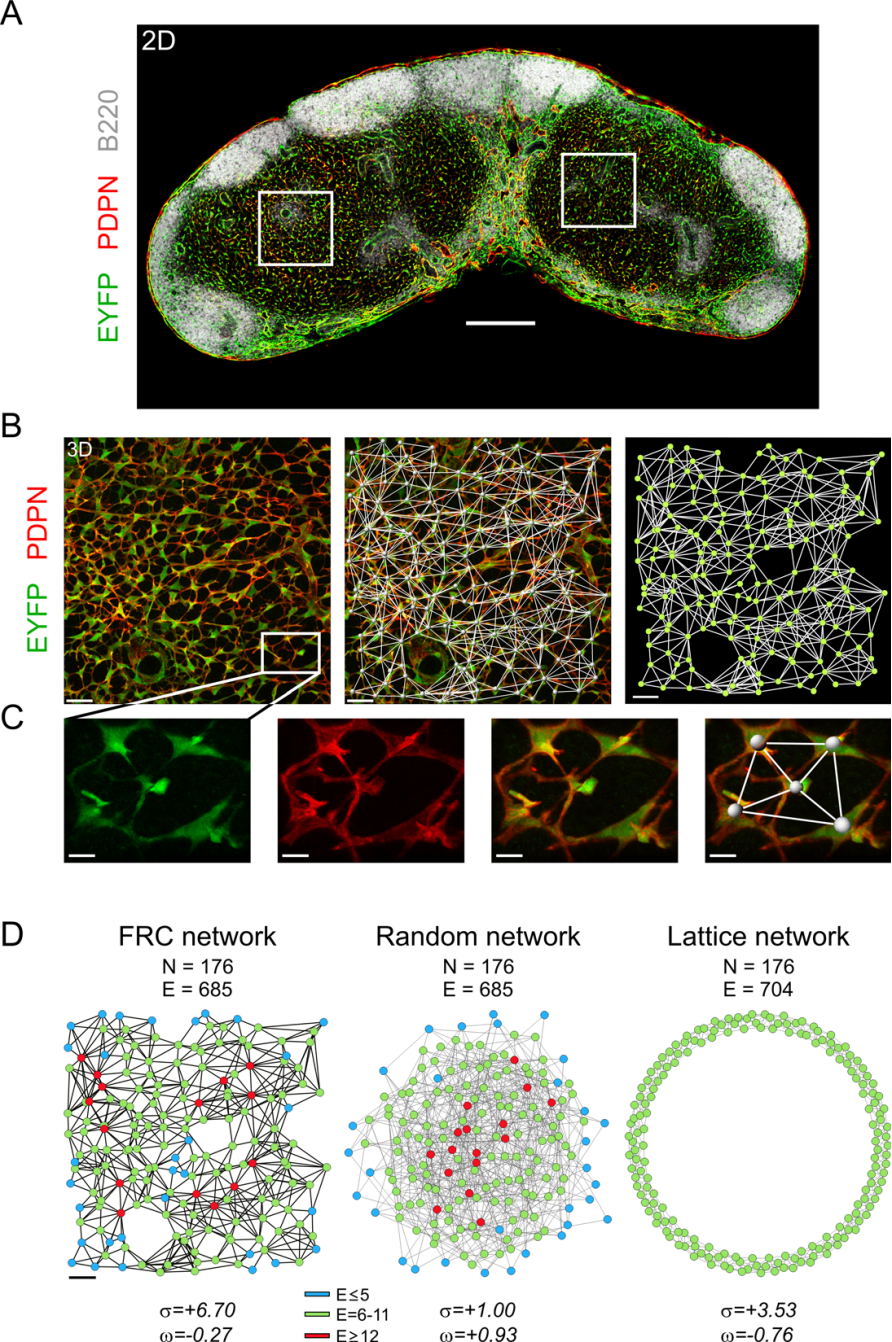
Assessing the topology of the T cell zone FRC network. (A) Overview 2-D image of an inguinal LN section from a naive adult *Ccl19*^*eyfp*^ mouse stained with antibodies against the indicated markers. Rectangles indicate representative T cell zones acquired with high-resolution confocal microscopy. (B) Representative 3-D Z-stack indicating the T cell zone FRCs (left panel), merged with FRC network (middle panel) and the network representation (right panel) with nodes (FRCs) and edges (physical connections). Size of T cell zone image: 304 x 304 x 32 μm. (C) Zoom-in area of single FRCs from (B, left panel) with signals for EYFP, PDPN, merged, and network representation, respectively. (D) Representative FRC network from (B, right panel). The equivalent random network was constructed using the Erdos-Renyi model, and the regular ring lattice network was constructed with eight edges for every node (FRC network median). Lattice and random networks are shown in Kamada-Kawai representation, while the FRC network is arranged in the real coordinate system of the LN T cell zone. N denotes the number of nodes, and E denotes the number of edges for each network. Small-world parameters σ and ω are shown below. The color legend represents number of edges per node. Data are representative of six mice from two independent experiments. Scale bars represent 300 μm (A), 30 μm (B, D), and 10 μm (C).

The different microenvironments of the LN, e.g., T or B cell zones or the subcapsular region, are built by distinct FRC subpopulations [10]. Importantly, all LN FRCs can be specifically targeted in vivo using the *Ccl19-cre* mouse model (Fig 1A), whilst transgene expression is absent in hematopoietic cells [5] including CD11c^+^MHCII^high^ DCs (not shown). For the structural network analysis, we have focused on the classical podoplanin (PDPN)-expressing T cell zone FRCs that orchestrate the interaction of DCs and T cells [31] and provide important survival factors for T cells, such as interleukin 7 (IL-7) [6,32]. Three-dimensional reconstruction of high-resolution confocal microscopy Z-stacks covering a volume of 304 x 304 x 32 μm was applied to analyze the FRC network structure (Fig 1B) by defining nodes as the enhanced yellow fluorescent protein (EYFP)-positive FRC centers of mass and edges as physical connections between adjacent FRCs (Fig 1C and S1 Video).

Small-world networks exhibit the intrinsic property that most nodes can be reached from every other node by a small number of steps, even though most nodes are not direct neighbors. This enables small-world networks with fast and efficient information transfer, which is characterized by small shortest path lengths (node-to-node distances). These networks also exhibit high capacity for clustering (i.e., connectivity between neighboring nodes), which is strikingly different from random networks in which all nodes have the same probability of containing an edge. Thus, a specific network can be classified as a small-world network by comparing network-level statistics to equivalent random and lattice networks. Moreover, small-worldness can be described by the σ and ω parameters (see S1 Table), which classify a network as small world if σ > 1 and ω ≈ 0 (range −0.5 to +0.5) [33–35]. Accordingly, random networks will show σ ≈ 1 and positive 0 < ω < 1, while lattice networks will have σ > 1 like small-world networks but negative −1 < ω < 0. As shown in Fig 1D (left panel), a representative FRC network sample contained 176 nodes (N) and 685 edges (E) with σ and ω values of +6.7 and −0.27, respectively. Note that network connectivity is color coded with highly connected nodes (E ≥ 12) depicted in red. The equivalent random network with the same number of nodes and edges as the FRC network has both σ = 1 and ω = 0.93 positive (Fig 1D, middle). A regular ring lattice network with the same number of nodes and eight edges per node connecting to nearest neighbors fulfills the condition for small-worldness with σ = 3.53, while the negative ω = −0.76 identifies the lattice structure, as expected (Fig 1D, right). This initial network analysis with σ = 6.128 ± 0.659 and ω = −0.308 ± 0.069 (*n* = 6 mice) indicates that FRCs of the T cell zone form a small-world network with lattice-like properties.

### Efficient Restoration of FRC Network Properties after Complete Node Removal

**Fig 2:**
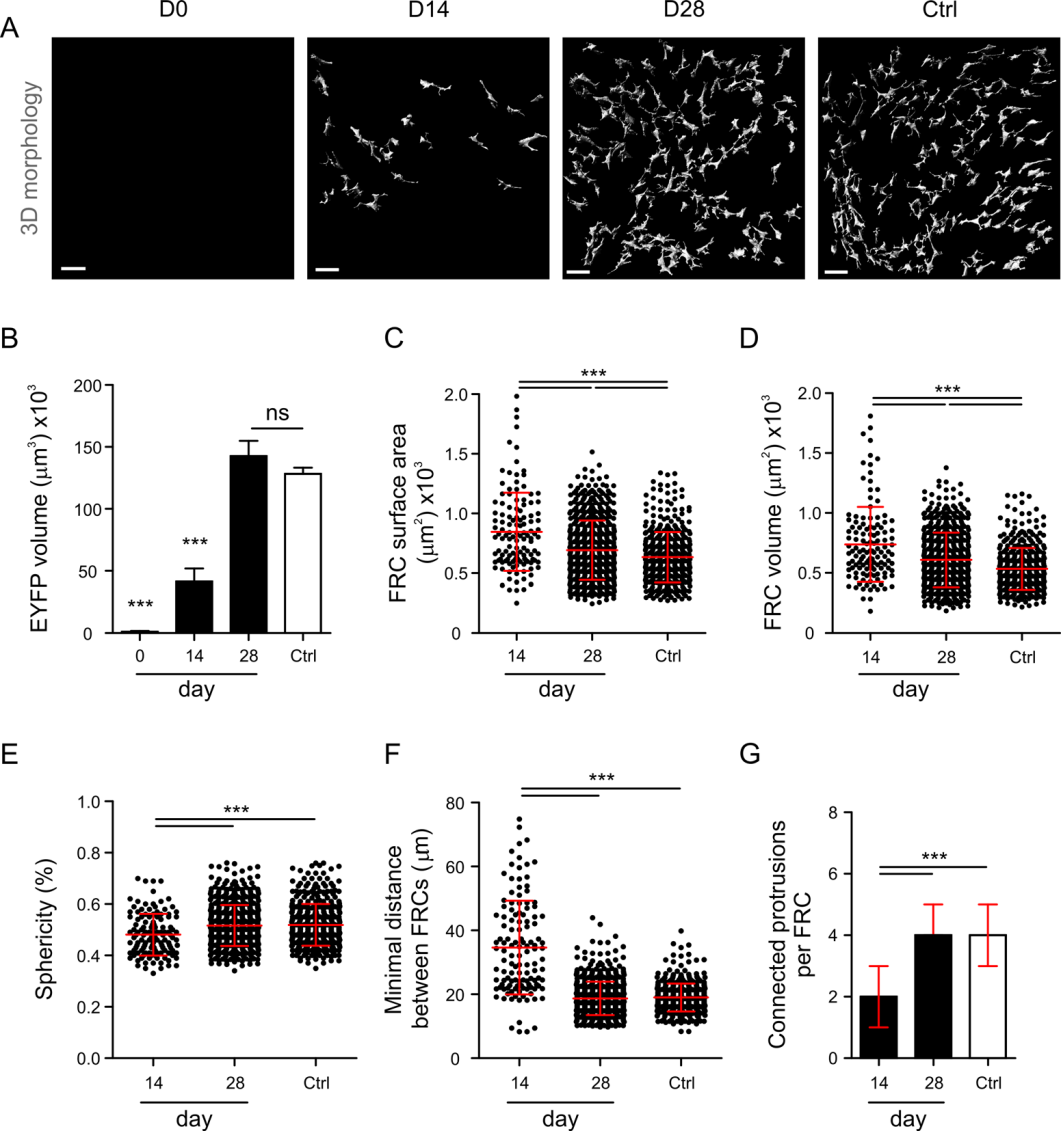
Changes in FRC morphology following diphtheria toxin (DT)-mediated ablation. (A) Three-dimensional single-cell reconstruction of the T cell zone FRC network in *Ccl19*^*eyfp/idtr*^ mice at indicated time points after two intraperitoneal (IP) injections of 8 ng/g DT or phosphate-buffered saline (PBS)-treated controls. Scale bars represent 30 µm. (B) Global morphological analysis of the total FRC network volume from the 3-D-reconstructed EYFP channel. (C–G) Single-cell analysis of FRC surface area (C), volume (D), sphericity (E), minimal distance between FRCs (F), and connected protrusions per FRC (G). Each dot represents a measurement for a single FRC. Data represent mean ± standard deviation (SD) (B–F) and median ± interquartile range (IQR) (G) for 3–5 mice per group. * *p* < 0.05, ** *p* < 0.01, *** *p* < 0.001 (one-way ANOVA with Tukey’s post-test [B–F] or Kruskal-Wallis test with Dunn’s post-test [G]). ns, not significant.

**Fig 3:**
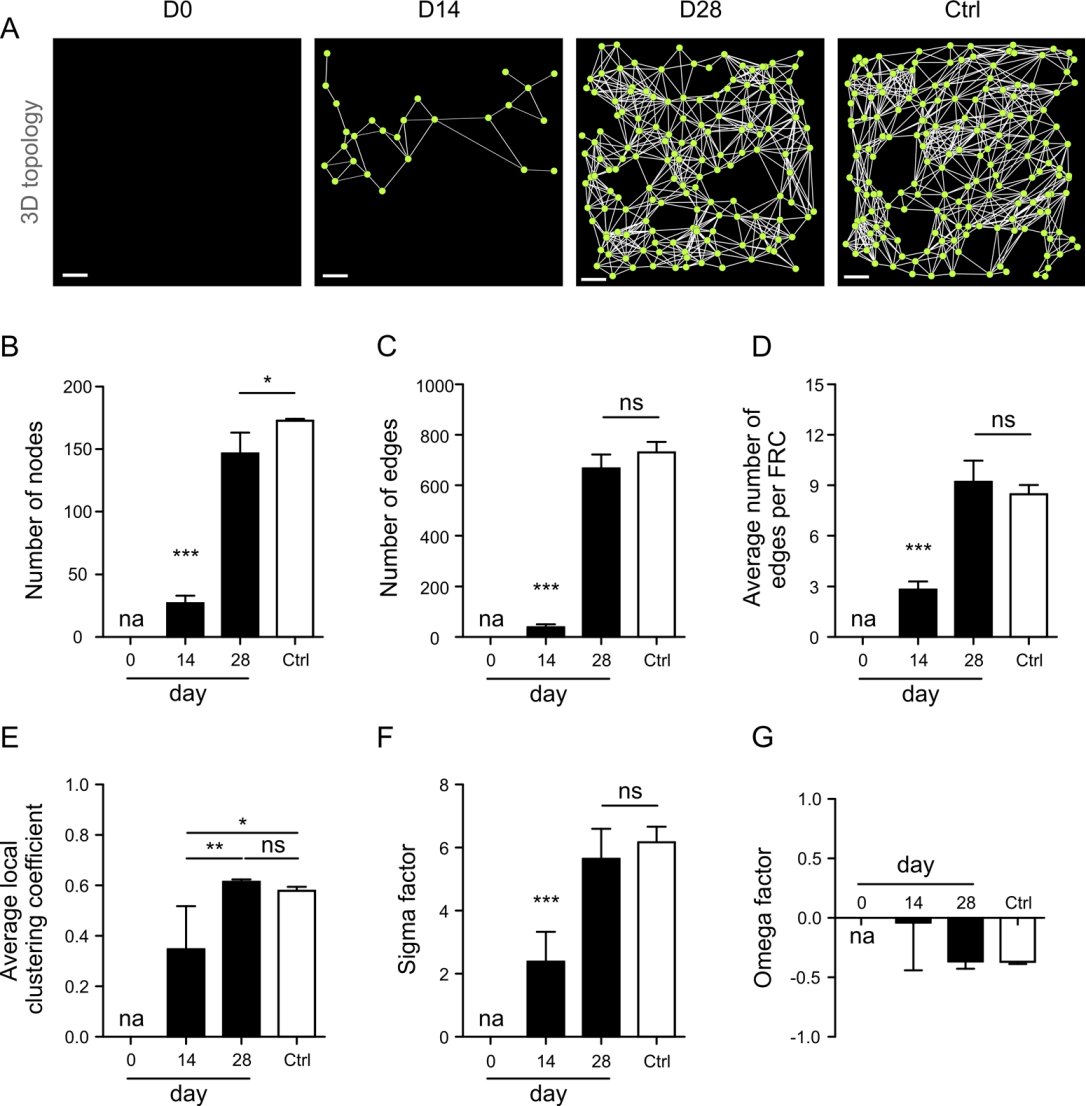
FRC network restoration following DT-mediated ablation. (A) Representative FRC network analysis using nodes as single FRC centers of mass and edges as physical connections between adjacent cells. Scale bars represent 30 µm. (B–G) Topological network analysis of the FRC network at indicated time points after two IP injections of 8 ng/g DT or PBS-treated controls. Network-level statistics shown are total number of nodes (B) and edges (C) in the network, average number of edges per FRC (D), average local clustering coefficient (E), and small-world parameters σ (F) and ω (G). Data represent mean ± SD for 3–5 mice per group. * *p* < 0.05, ** *p* < 0.01, *** *p* < 0.001 (one-way ANOVA with Tukey’s post-test). ns, not significant; na, not applicable.

Since the functional properties of a network are determined by its structure [36], we assessed first whether the FRC network structure is hardwired and will be reestablished after removal of all nodes. To this end, we used specific FRC ablation in mice that express both the diphtheria toxin receptor (DTR) and EYFP under the control of the *Ccl19* promoter (*Ccl19*^*eyfp/idtr*^) [7]. To achieve complete ablation of FRCs at the start of the experiment (i.e., day 0), 8 ng DT per g body weight were injected intraperitoneally on days −5 and −3 (S1A Fig). PDPN^+^EYFP^+^ FRCs in T cell zones were removed, while PDPN expression in and around high endothelial venules was partially maintained (S1B and S1C Fig). The FRC network was partially restored on day 14 (Fig 2A, S1B and S1C Fig), with approximately 32% of the EYFP volume restored (Fig 2B). Importantly, the FRC network had been rebuilt on day 28 to an extent that was indistinguishable from controls (Fig 2A and 2B, S1B and S1C Fig). However, basic single-cell parameters, namely FRC surface area (Fig 2C) and volume (Fig 2D), had not yet reached the levels of controls, while other morphological parameters such as cell sphericity had returned to normal values (Fig 2E). Moreover, the FRC network had reached the original cell distribution with identical intercellular distances (Fig 2F) and number of connected protrusions per cell (Fig 2G), suggesting that the FRC network structure can be restored from scratch within approximately 4 wk. Indeed, topological network analysis (Fig 3A) confirmed that essential network parameters such as the number of nodes (Fig 3B) and edges (Fig 3C) had almost completely returned to the levels of controls. Further network properties such as the number of edges per FRC (Fig 3D) and the local clustering coefficient (Fig 3E) had been restored as well. Likewise, small-worldness, as determined by the σ (Fig 3F) and ω factors (Fig 3G), was maintained after 28 d, indicating that the FRC network small-world structure is an imprinted trait of the LN infrastructure.

### Distinct Thresholds Govern FRC Network Structural Integrity

**Fig 4:**
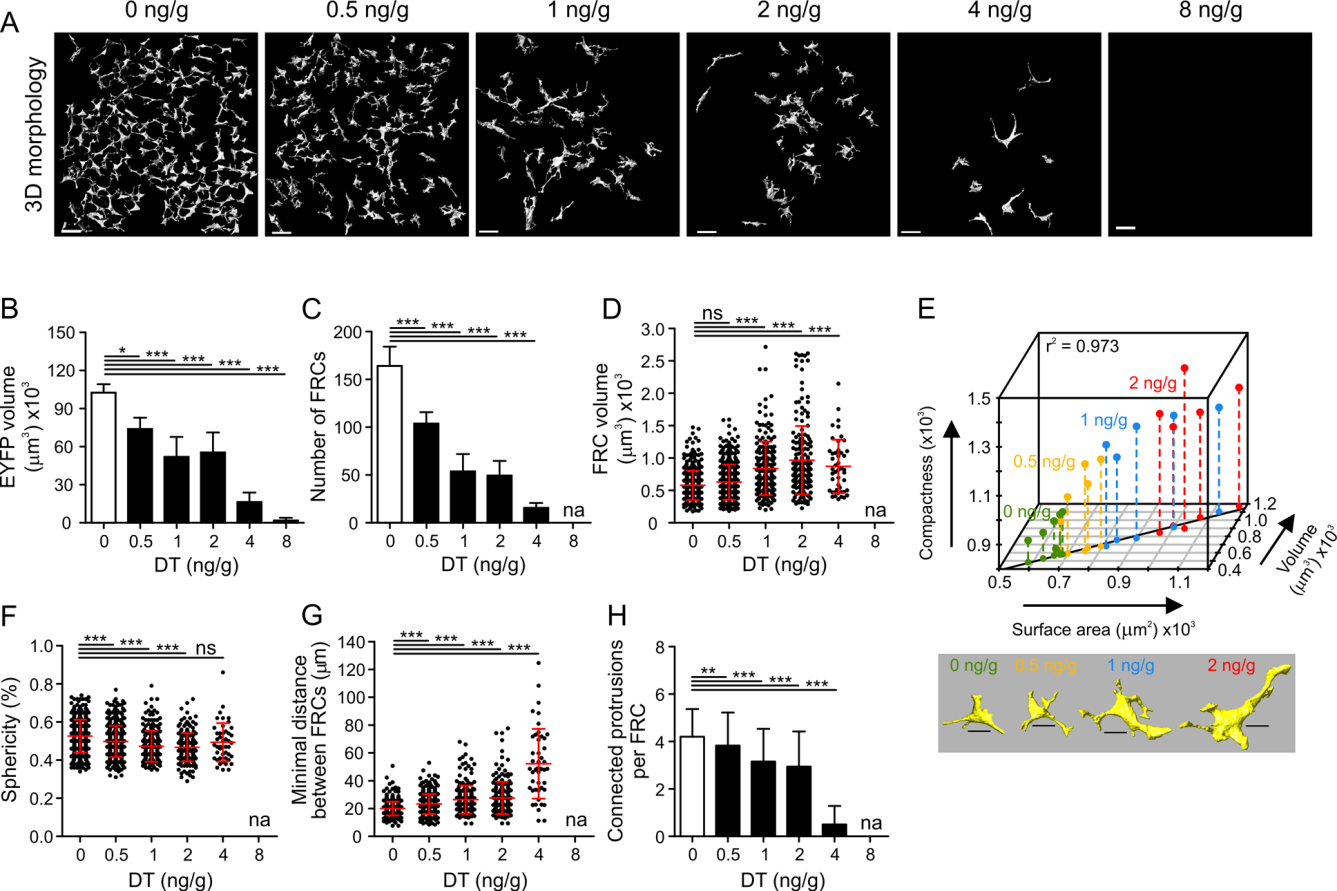
Alterations in FRC morphology following partial FRC ablation. (A) Representative 3-D single-cell reconstructions of the T cell zone FRC network in *Ccl19*^*eyfp/idtr*^ mice injected twice IP with indicated doses of DT. (B) Total volume of the EYFP^+^ T cell zone FRC network for indicated doses of DT. (C) Number of single FRCs per acquired T cell zone for indicated doses of DT. (D–H) Single-cell analysis of FRC volume (D); correlation of surface area, volume, and compactness (E); sphericity (F); minimal distance between FRCs (G); and connected protrusions per FRC (H). Values in (B–C) represent mean ± SD for each T cell zone FRC dataset and in (D, F–H) represent mean ± SD for each single FRC for 3–6 mice per group from two independent experiments. Vertical lines in the 3-D plot (E) represent projections on the bottom 2-D plane. The line on the 2-D plane represents a linear regression model for surface area and volume with indicated Pearson correlation coefficient r^2^ = 0.973, *p* = 2.71 x 10^−16^ (Fisher’s F test). Images below are representative 3-D reconstructions of FRCs for indicated doses of DT. Scale bars represent 30 μm (A) and 10 μm (E). * *p* < 0.05, ** *p* < 0.01, *** *p* < 0.001 (one-way ANOVA with Tukey’s post-test [B–D and F–G] or Kruskal-Wallis test with Dunn’s post-test [H]). ns, not significant; na, not applicable.

**Fig 5:**
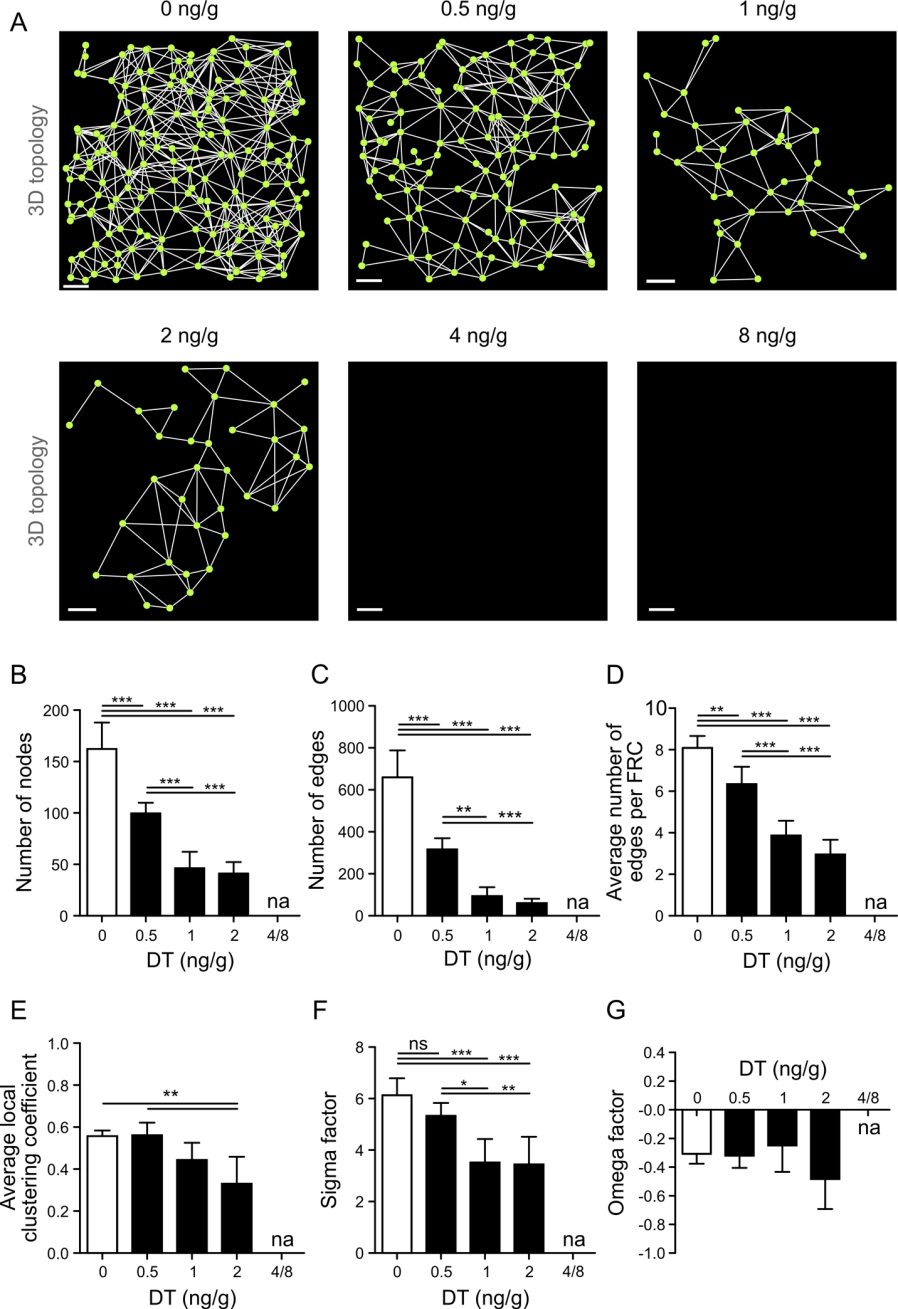
Gradual FRC ablation reveals thresholds for FRC network integrity. (A) Topological analysis of the FRC network in *Ccl19*^*eyfp/idtr*^ mice injected twice IP with indicated doses of DT. Scale bars represent 30 μm. (B–G) Network analysis of the FRC network at indicated doses of DT. Network-level statistics shown are total number of nodes (B) and edges (C) in the network, average number of edges per FRC (D), average local clustering coefficient (E), and small-world parameters σ (F) and ω (G). Data represent mean ± SD for 3–6 mice per group from two independent experiments. * *p* < 0.05, ** *p* < 0.01, *** *p* < 0.001 (one-way ANOVA with Tukey’s post-test). ns, not significant; na, not applicable.

The next set of experiments was performed to determine the structural stability of the FRC network under conditions of partial removal of nodes. Graded doses of DT were applied, and FRC morphology and topology were assessed. As shown in Fig 4A and S2 Fig, application of 0.5 ng/g DT resulted in moderate FRC ablation, while doses above 2 ng/g resulted in substantial damage to the FRC network. Global morphological analysis confirmed the drastic effect of DT doses >2 ng/g on the EYFP^+^ cell population (Fig 4B). FRC numbers decreased by 37%, 67%, 70%, 91%, and 100% in mice treated with 0.5, 1, 2, 4, and 8 ng/g DT, respectively (Fig 4C). Single-cell analysis revealed a steady increase in FRC volume with higher DT doses (Fig 4D). Moreover, other cellular parameters such as compactness and surface area also increased with decreasing FRC density (Fig 4E), while FRC sphericity was decreasing (Fig 4F). It is most likely that these morphological changes are a consequence of FRC relaxation by which the cells compensate for the loss of neighboring cells or the need to cover more space [7,10,37]. Interestingly, minimal distances between neighboring FRCs substantially increased when cell loss was higher than 70% (Fig 4G). Moreover, connectivity between FRCs was almost completely lost at DT doses >2 ng/g (Fig 4H), suggesting that the FRC network had been substantially disintegrated. Topological network analysis confirmed that a distinct threshold for FRC network integrity exists, as the network structure was destroyed when more than 70% of the cells were ablated (Fig 5A). Interestingly, the number of nodes (Fig 5B) and edges (Fig 5C) dropped substantially when only 37% of the FRCs were ablated. However, other network parameters such as the number of edges per FRC (Fig 5D) and the local clustering coefficient (Fig 5E) were not profoundly altered at the DT dose of 0.5 ng/g. Likewise, small-worldness as determined by the σ factor was not significantly affected when the FRC network was mildly perturbed by the low-dose DT injection, while >50% FRC loss (i.e., DT doses of 1 ng/g and 2 ng/g) resulted in a substantial change in this network parameter (Fig 5F). It appears that the ω factor is not sensitive to strong alterations in the FRC network introduced by partial node removal (Fig 5G), suggesting that the FRC network remains preferentially latticed. Nevertheless, the topological analysis based on increasing FRC ablation indicates that the essential FRC network features remain stable when <40% of the cells are removed, while an ablation of >70% of FRCs results in complete network failure.

### In Silico Prediction of the FRC Network Topological Robustness

**Fig 6:**
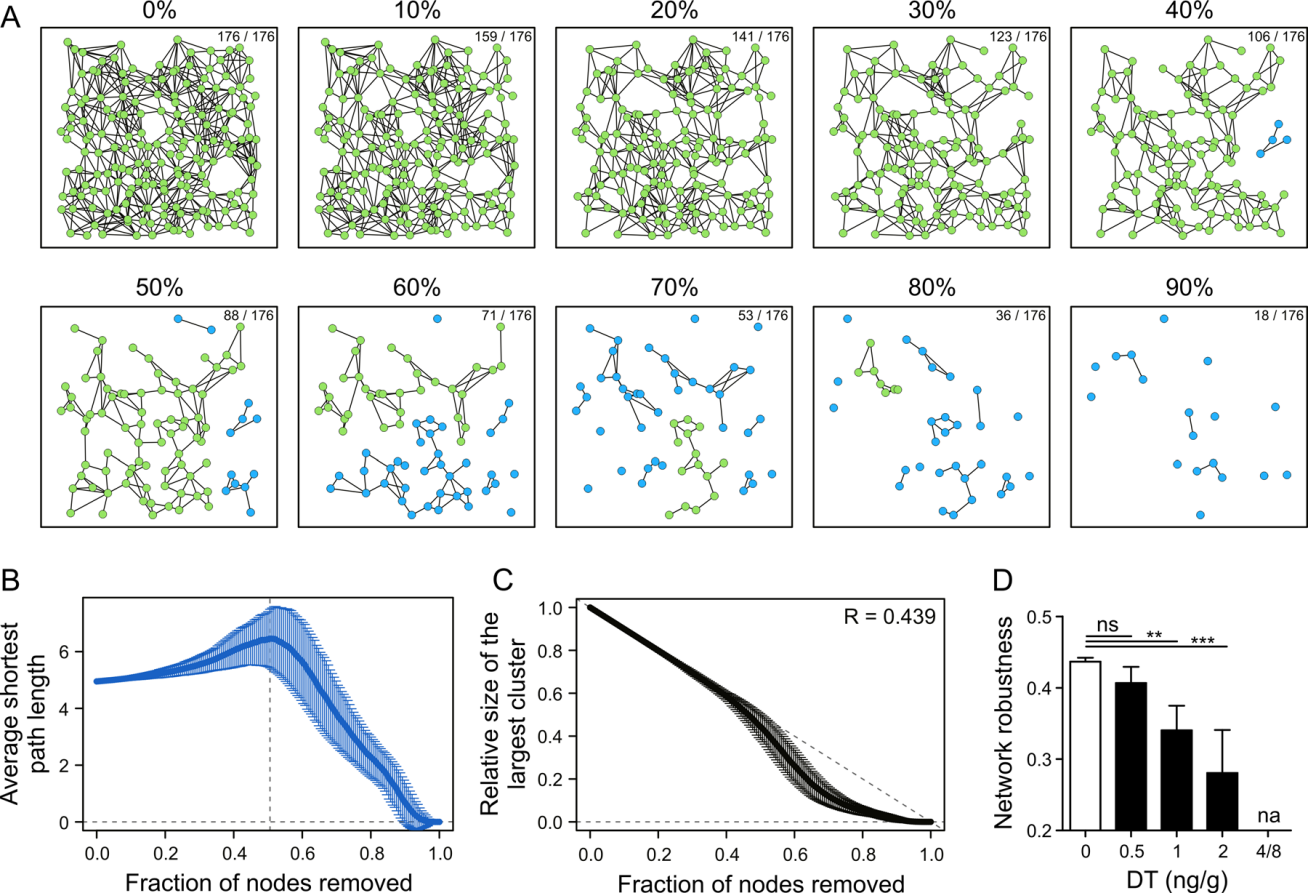
Graph theory-based analysis of the FRC network topological robustness. (A) In silico perturbation analysis of a representative FRC network from PBS-treated control mice by random node removal for one simulation. Each image denotes the FRC network in a real coordinate system of the LN T cell zone at indicated fractions of nodes randomly removed. The number of nodes remaining and the starting number of nodes are indicated in the top right of each image. Green nodes represent the largest connected cluster, and blue nodes represent fragmented clusters. See S2 Video for the full simulation. (B) Average shortest path length versus fraction of nodes removed. The dashed line represents fraction of nodes removed for the maximal value of average shortest path length, i.e., the network threshold point. (C) Relative size of the largest cluster compared to the size of the starting network at 0% versus fraction of nodes removed. The indicated value in the top right denotes estimated network robustness R. The dashed line represents the minimal damage line. Data represent mean ± SD over 1,000 simulations of random node removals for a representative FRC network (*n* = 6 mice from two independent experiments). (D) Network robustness R values for FRC networks at indicated doses of DT. Data represent mean ± SD for 3–6 mice per group from two independent experiments. * *p* < 0.05, ** *p* < 0.01, *** *p* < 0.001 (one-way ANOVA with Tukey’s post-test). ns, not significant; na, not applicable.

Network failure occurs when nodes lose their function in a random fashion or as a consequence of targeted destruction of particular nodes. Importantly, both network topology and the nature of node loss determine the robustness of the network [14]. Here, we reasoned that rapid loss of LN FRCs, e.g., during a viral infection [4], occurs in a random fashion. Likewise, we considered DT-mediated removal of FRCs in the *Ccl19*^*idtr*^ model as arbitrary. Therefore, we first performed an in silico perturbation analysis by sequentially removing nodes from the FRC network model in a randomized manner (S2 Video). Network fragmentation kinetics were followed during removal of nodes and their associated connections, in order to evaluate the topological properties of the residual network at each step (Fig 6A). As nodes are removed, network fragments are generated (blue) that are disconnected from the largest cluster of nodes (green) (Fig 6A). For each 3-D-reconstructed FRC network, 1,000 simulations of randomized node removal were performed (Fig 6B and 6C). These datasets permitted estimation of the network integrity threshold across all fractions of nodes removed, corresponding to the maximal value of average shortest path length of the largest cluster (Fig 6B). The analysis revealed that the network started to lose the characteristic path length when approximately 50% of the nodes were removed (Fig 6B). Note that 50% node removal corresponds to FRC ablation with DT doses between 0.5 and 1 ng/g DT, which lead to reduction of FRC numbers by 37% and 67%, respectively (Fig 4C). In addition, we determined the fragmentation curve as the relative size of the largest cluster compared to the size of the starting network and fraction of nodes removed (Fig 6C). In this type of analysis, a network will have higher robustness the closer the curve is to the minimal damage line (Fig 6C, dashed line). The perturbation analysis demonstrated that the FRC network exhibits high robustness to random node removal, indicated by a robustness value R of 0.439 (Fig 6C). Note that the estimated network robustness ranges between maximal vulnerability (R = 0) and maximal robustness (R = 0.5). The network robustness for all phosphate-buffered saline (PBS)-treated controls was estimated 0.437 ± 0.005, *n* = 6 mice (Fig 6D). Importantly, the topological model predicts that network robustness is not significantly reduced when 37% of the FRCs are ablated, while ablation of >50% of FRCs, i.e., at doses of 1 and 2 ng/g DT, will lead to a significant reduction of network robustness (Fig 6D). Collectively, the in silico model predicts that the FRC network displays significant topological robustness against random node removal and is able to tolerate up to half of the network being destroyed.

### Impact of Altered FRC Network Topology on LN Functionality

**Fig 7:**
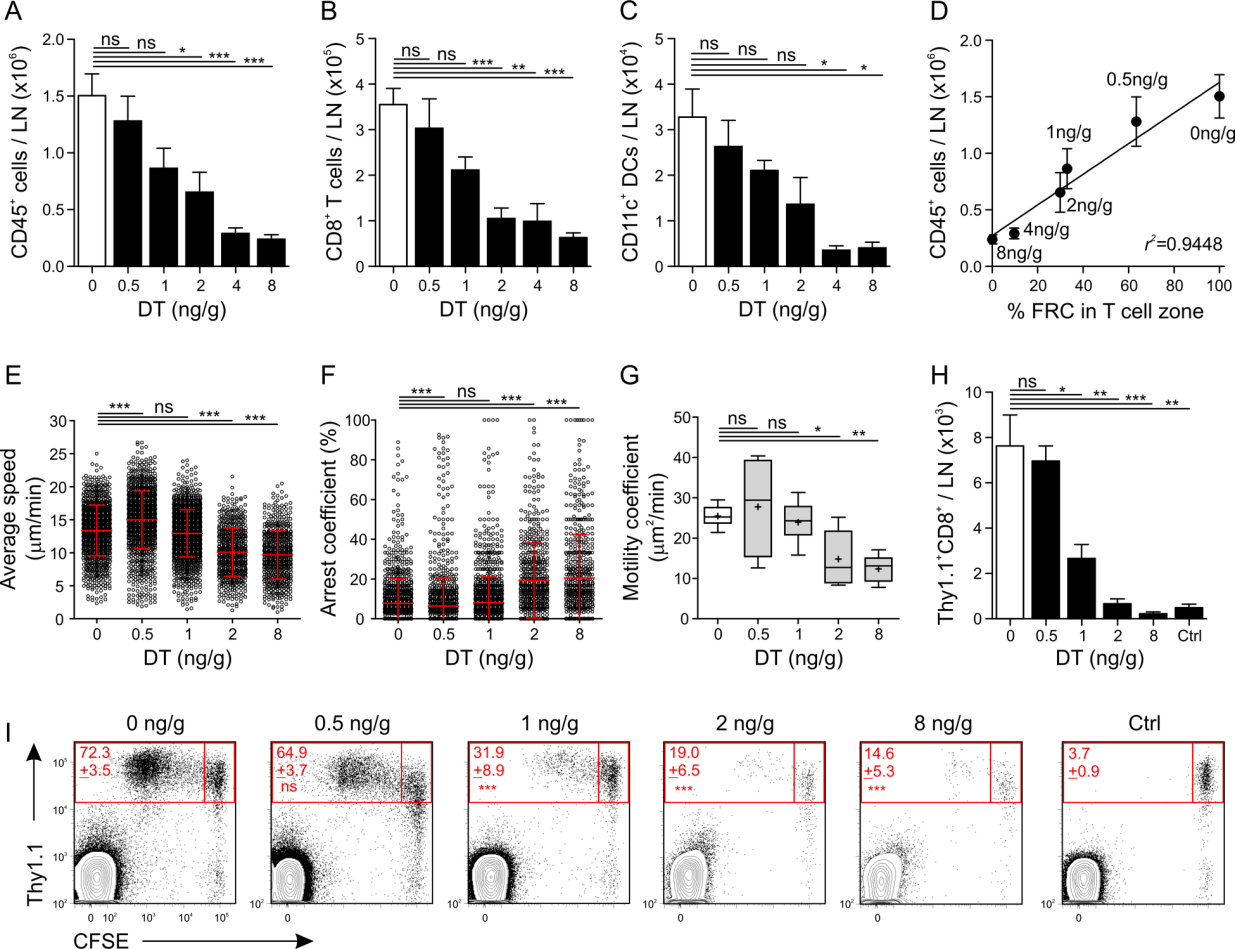
Impairment of LN functionality following FRC ablation. LN cellularity as determined by flow cytometry with total numbers of CD45^+^ hematopoietic cells (A), CD8^+^ T cells (B), and CD11c^+^ DCs (C) in *Ccl19*^*idtr*^ mice injected twice IP with the indicated doses of DT. (D) Correlation between CD45^+^ cells and FRCs remaining in the LN for indicated doses of DT with Pearson correlation coefficient r^2^ = 0.9448, *p* = 0.00117 (Fisher’s F test). (E–G) Two-photon microscopy analysis of adoptively transferred CD8^+^ T cells into *Ccl19*^*idtr*^ mice injected IP with indicated doses of DT. The migration parameters analyzed include average cell speed (E), cell arrest coefficient (F), and motility coefficient (G). (H) Total numbers of transferred TCR-transgenic Thy1.1^+^CD8^+^ T cells in *Ccl19*^*idtr*^ LNs at indicated doses of DT. (I) Flow cytometric analysis of CD8^+^ T cell activation in *Ccl19*^*idtr*^ LNs on day 3 post immunization with DC-targeting viral particles. Numbers indicate mean percentage ± standard error of the mean (SEM) of proliferating Thy1.1^+^ cells of the whole Thy1.1^+^ population. Indicated *p*-values represent comparison to the 0 ng/g group. Controls indicate PBS-treated mice without viral particles. Representative experiment for 3–6 mice per group from three independent experiments. Data represent mean ± SEM for 3–20 mice per group from three independent experiments (A–D, H). Data represent mean ± SD (E–F) or median ± range (G) for 5–10 datasets from 2–3 mice per group from two independent experiments. Plus “+” indicates mean. * *p* < 0.05, ** *p* < 0.01, *** *p* < 0.001 (one-way ANOVA with Tukey’s post-test [A–C, H–I] and Benferroni’s post-test [G] or Kruskal-Wallis test with Dunn’s post-test [E–F]). ns, not significant.

LNs control distribution of immune cells in the body by attracting lymphocytes and myeloid cells via afferent lymph and blood. In addition, cellular content in the LN is influenced by cell release into efferent lymph [38]. To assess how LN FRCs impinge on the immune cell content of LNs, we determined the numbers of CD45^+^ hematopoietic cells (Fig 7A), CD8^+^ T cells (Fig 7B), and CD11c^+^ DCs (Fig 7C) following graded FRC ablation. Interestingly, ablation efficacy <40% (i.e., at 0.5 ng/g DT) did not lead to significantly reduced cell numbers, while FRC ablation above 70% precipitated profound changes in LN cellularity (Fig 7A–7C, S3A and S3B Fig). Plotting FRC density against hematopoietic cell numbers under conditions of graded FRC depletion revealed a clear dependence of immune cell aggregation in LNs on FRC network integrity (Fig 7D). Next, we determined whether and to what extent intranodal T cell migration depends on the presence of FRCs. To this end, TCR transgenic CD8^+^ T cells [39] were adoptively transferred into DT-treated *Ccl19*^*idtr*^ mice, and T cell behavior was assessed by two-photon microscopy (S3 and S4 Videos). Cell tracking analysis revealed comparable T cell speeds and arrest coefficients with DT doses of ≤1 ng/g. In contrast, a significant decrease in T cell speeds was observed at DT doses of ≥2 ng/g, with a concomitant increase in cell arrest (Fig 7E and 7F). Accordingly, T cell tracks exhibited decreased motility coefficients (Fig 7G), a measure of scanning efficacy, and decreased meandering index (S3C Fig), a measure of movement straightness, when >70% of FRCs were ablated. Overall, analysis of these data indicated that substantial changes in intranodal T cell migration occurred when >70% of FRCs were lost, i.e., at DT doses of ≥2 ng/g. To assess how FRCs affect DC-mediated activation of antiviral CD8^+^ T cells, we resorted to a viral vector system that facilitates exclusive in vivo targeting of DCs [40,41]. Propagation-deficient coronavirus particles were injected subcutaneously into FRC-depleted mice, and the activation of antiviral CD8^+^ T cells was assessed in draining LNs. As shown in S3D and S3E Fig, T cell receptor transgenic Spiky cells were closely associated with the FRC network. Strikingly, T cell expansion was highly dependent on the presence of an intact FRC network because an ablation of >50% of FRCs resulted in an almost complete failure to expand the antiviral T cell population (Fig 7H). Labeling of the CD8^+^ T cells with an intracellular dye revealed that proliferation of the cells was substantially affected at DT doses of ≥1 ng/g (Fig 7I), suggesting that activation of naïve CD8^+^ T cells by DCs can be maintained as long as approximately 50% of the FRC network remains intact.

The high correlation between topology and biological function as shown in Fig 7D (r^2^ = 0.9448, *p* = 0.00117) prompted us to further assess overall correlation between FRC morphology, topology, and function. As shown in the heat map in S4 Fig, most parameters are highly correlated with increasing doses of DT (Pearson r > 0.8), indicating that they are decreasing with declining FRC numbers. Four parameters showed high anticorrelation, namely arrest coefficient, cell surface area, volume, and cell-to-cell distances, due to their increase with decreasing numbers of FRCs. Only the omega factor did not significantly correlate with any other parameter as it is not sensitive to DT treatment (Fig 5G). Overall, this analysis demonstrates the intricate connection between LN functionality and FRC topology.

## Discussion

Phenotypical characteristics of biological systems arise from complex interactions between cells that are orchestrated in a highly organized spatial and timely manner. Hence, it is a major challenge to understand the structure and dynamics of cellular networks and infer the function of particular tissues and organs. Results of the present study show that the physical scaffold of LNs formed by FRCs is critical for the maintenance of LN functionality. It is conceivable that the structure of the FRC network optimizes area/volume scanning by T cells by improving accessibility to distant regions [42]. Moreover, recent findings suggest that FRCs regulate the motility of DCs through PDPN-(C-type lectin-like receptor 2) CLEC2 interaction [43]. Our results are in line with a previous study that demonstrated profound effects of complete FRC network ablation on T and B cell activation [7]. However, the complete destruction or ablation of components does not reveal the extensive complexity of a system and the role of specific components in its robust performance. In particular, global systems parameters such as topological organization and robustness need to be considered in order to design strategies for system modulation or regeneration.

The theory of complex networks, i.e., graph theory, offers a novel conceptual framework for biological systems and can be used as a powerful tool to dissect the quantifiable patterns of interaction between cells and the structure–function relationship of biological systems [13,44]. The present graph theory-based analysis revealed that LN FRCs form a small-world network with lattice-like properties. These properties were fully restored following complete removal of all FRCs, indicating that the basic FRC network topology with substantial connectivity and high capacity for clustering is an imprinted structural trait. It is possible that FRC network regeneration is guided by collagen fibers that are produced by FRCs [45] and remain visible after FRC ablation [7]. Assessing the interdependence between FRCs and the collagen fiber network will reveal further basic principles of LN organization and functionality.

Our topological analysis was restricted to representative samples of T cell zone FRCs, mainly because a substantial part of the LN FRCs can be found in multilayered sheaths around blood and lymph vessels [10]. Even high-resolution confocal microscopy did not provide the means to separate single perivascular FRCs and to assess their morphology and topology. However, the methodology applied in our study is suitable to assess FRC network topology in B cell zones where distinct subsets such as C-X-C motif chemokine 12 (CXCL12)-producing FRCs [9] control B cell migration. We envision that utilization of extended-volume imaging systems [46] or selective plane illumination microscopy [47] at high resolution will provide means to achieve an extended topological analysis of the LN FRC network. Nevertheless, the topological model based on samples of the T cell zone FRC network, as applied here, predicted with high accuracy the functional consequences of FRC loss, indicating that the sample area was adequately large to infer the behavior of the whole network. The morphological and topological parameters generated here will help to further advance development of mathematical LN models and could stimulate further research in modeling cell migration and fluid transport phenomena in other SLOs.

Several modeling approaches have been described that focus on the description of processes that occur in LN subcompartments such as DC-driven T cell migration [48] or differentiation of Th cell subsets [49]. However, in order to obtain a more holistic view on LN functionality, the complexity of multilayered processes needs to be captured in mathematical models, an endeavor that requires representation of the whole LN [50]. Simple models have addressed this challenge by symbolizing the basic structural elements (either in 2-D or 3-D) as a regular orthogonal lattice [51,52]. Such agent-based models can describe the behavior of a variety of different cell types under the provision of distinct rules for their interaction. In addition, hybrid approaches have utilized ellipsoid, 3-D lattice models combined with agent-based modeling of immune cell interaction that facilitated simulation of antigen encounter under inflammatory conditions [53]. Clearly, steadily increasing computer power combined with novel imaging techniques provides a wealth of information describing immune cellular location [54] and principles of structural organization such as the LN vasculature [46,55]. Hence, it will be possible to generate extended mathematical models that describe multiple, interdependent immune reactions in LNs based on realistic geometry. Our study demonstrates that graph theory-based analysis of LN structures such as the FRC network not only provides important information on basic organization principles but also facilitates accurate prediction for the outcome of immune reactions. This suggests that the R index of the FRC network can be considered as a biologically relevant and consistent measure of robustness with global functional implications in the immune system. The suitability of this approach for in-depth analysis of critical biological processes has been shown in studies on neuronal networks [44]. Interestingly, neurons form—as FRCs—physically connected small-world networks that determine the function of the whole organ [56]. It is possible that these physical, non-random networks might have developed under evolutionary pressure to establish their structure and achieve optimal functionality.

Overall, we anticipate that implementation of graph theory-based approaches in the investigation of those cellular elements that determine LN structure and functionality will fill the gaps in the understanding of critical immune processes. Moreover, generating improved mathematical models that permit prediction of complex system behavior will promote the development of rationally designed immune therapies and impinge on therapeutic intervention in diseases with involvement of immune system components.

## Materials and Methods

### Mice and Selective FRC Ablation

C57BL/6N (B6) mice were purchased from Charles River Laboratories. BAC-transgenic C57BL/6N-Tg(Ccl19-Cre)489Biat (*Ccl19-Cre*) [5] crossed to iDTR mice [57] and TCR transgenic mouse strain C57BL/6N-Tg(Tcra,Tcrb)577Biat (Spiky) [39] have been previously described. DT was applied at days −5 and −3 via IP injection at the indicated doses following established protocols [7]. All mice were maintained in individually ventilated cages and were used at the age of 6 to 9 wk. Experiments were performed in accordance with federal and cantonal guidelines (Tierschutzgesetz) under the permission numbers SG13/05 and SG13/04 following review and approval by the Veterinary Office of the Canton of St. Gallen and under permission BE48/11 granted by the Veterinary Office of the Canton of Bern.

### LN Cellularity

For flow cytometric analysis of LN cellularity, inguinal LNs from individual mice were pooled and digested on 37°C in RPMI containing 2% FCS, 20 mM Hepes (all from Lonza), 1 mg/ml Collagenase Type P (Sigma-Aldrich), and 25 μg/ml DNaseI (AppliChem) for 20 min. After enzymatic digestion, cell suspensions were washed with PBS and stained using the following antibodies: CD3-PE (BD Bioscience), CD8-PeCy7, CD4-FITC, CD45-APC-H7, MHCII-PE, CD11c-PeCy7, B220-APC (BioLegend). In flow cytometric analyses, 7-amino-actinomycin D (7AAD; Calbiochem) was used to discriminate dead cells. Samples were analyzed by flow cytometry using a FACSCanto flow cytometer (BD Biosciences); data were analyzed using FlowJo software (Tree Star).

### Antigen-Specific T Cell Responses

Single-cell suspensions from spleens were prepared by mechanical disruption of the organ and subjected to hypotonic red blood cell lysis. For in vivo proliferation, splenocytes were labeled using CFSE or intracellular dye Alexa-670 (Molecular Probes) according to the manufacturer’s protocol, and 2 x 10^7^ cells (corresponding to 2 x 10^6^ CD8^+^ TCR transgenic T cells) were transferred intravenously (IV) into FRC-depleted recipient mice. Twelve hours post adoptive transfer, the mice were subcutaneously injected with 3 x 10^6^ of non-replicating coronaviral particles in both flanks. A second injection of non-replicating coronaviral particles was performed 12 h following the first one. After 72 h from the first viral particle injection, inguinal LNs from individual mice were collected and analyzed using FACS.

### Immunohistochemistry and Morphometric 3-D Reconstruction Analysis

LNs were fixed overnight at 4°C in freshly prepared 4% paraformaldehyde (Merck-Millipore) under agitation and subsequently washed in PBS for one additional day. Fixed tissues were embedded in 4% low melting agarose (Invitrogen) in PBS and sectioned with a vibratome (VT-1200; Leica). Forty μm sections were blocked in PBS containing 10% FCS, 1 mg/ml anti-FcRγ (BD), and 0.1% Triton X-100 (Sigma-Aldrich). Sections were incubated overnight at 4°C with the following antibodies: anti-gp38/PDPN, anti-B220 (Biolegend), and anti-YFP (Clontech). Unconjugated antibodies were detected using Alexa-fluor labelled secondary antibodies (Jackson Immunotools). To visualize nuclei, sections were stained with 4′,6-diamidin-2-phenylindol (DAPI) (Sigma-Aldrich). Microscopic analysis was performed using a confocal microscope (LSM-710; Carl Zeiss), and the datasets were processed with ZEN 2010 software (Carl Zeiss).

Three-dimensional reconstructions of the T cell zone FRC network were performed using Imaris (Bitplane). One to two T cell zones were acquired per LN per mouse by confocal laser scanning microscopy. The total surface area and the volume of the EYFP^+^ FRC network were calculated using the Surfaces module by reconstructing the FRC network in 3-D with an automatic threshold for fluorescent intensities and surface area detail of 0.3 μm. In order to remove background noise and cell fragments, a volume filter <10 μm^3^ was used. Single-cell morphometric analysis was used to calculate morphological parameters for FRCs. Single FRCs were isolated as separate 3-D Surface objects by using the cutting tool in the middle of the connected protrusions. DAPI staining was utilized by masking it to the EYFP channel in order to identify cell nuclei belonging specifically to FRCs and determine number of FRCs per imaged T cell zone. FRCs that were cut at the dataset borders encompassing more than half of the central body and diving and apoptotic cells, as well as perivascular FRCs, were excluded from further analysis. Centers of mass for each FRC were calculated using the Surfaces module, and minimal distances between single FRCs were determined using the Spots module and "Spots to Spots Closest Distance" XTension in Imaris. Sphericity was calculated in Imaris, indicating how spherical a 3-D object is. The compactness measure was calculated as (area^3^/volume^2^), which is minimized by a sphere. Connected protrusions per FRC were determined by utilizing the EYFP and PDPN channels, counted before the first branching point and connected to another FRC. Detailed information about morphological parameters is available in S1 Table.

### FRC Network Topological Analysis

The topological model of the FRC network structure was created as an undirected, unweighted graph with no isolates in Imaris by defining nodes as the EYFP^+^ FRC centers of mass and edges as physical connections between neighboring FRCs. PDPN was utilized in order to visualize cell-to-cell connections more accurately. Topological analysis of the network was performed using the *igraph* package in R and RStudio. Small-world organization of the network was determined according to σ and ω parameters as described in [33–35]. For the calculation of the small-world parameters, the values of shortest path length and clustering coefficient were averaged over 100 realizations of an equivalent Erdos-Renyi random network for each FRC network dataset per mouse. Detailed information about topological parameters is available in S1 Table.

### FRC Network Perturbation and Robustness Analysis

Network perturbation analysis was performed using the *igraph* package and procedures as described in [14], in order to assess error tolerance by sequentially removing increasing number of nodes randomly from the network. Network robustness was estimated using the R parameter [58], and the threshold point was determined at the maximal value of the network average shortest path length as fractions of nodes are removed. Because of randomized node removal, the perturbation analysis was performed for 1,000 simulation runs for each FRC network dataset per mouse.

### Intravital Two-Photon Microscopy

3 x 10^6^ CellTracker Orange/CMTMR- or CellTracker Blue/CMAC-labelled P14 TCR transgenic T cells [59] were IV transferred into sex-matched *Ccl19*^*idtr*^ mice, which had received two injections of indicated dose of DT or PBS 3 and 5 d prior to T cell injection. Three to twenty-four hours after T cell injection, the right popliteal LNs of the recipient mice were surgically exposed as described previously [60]. Prior to image acquisition, 10 μg of AlexaFluor 633-labeled Meca79 antibody was injected IV in order to label HEVs. Each image sequence lasted for 30 min. Acquired 3-D time-lapse images were tracked using Imaris software with Spot and ImarisTrack function. Average single cell speeds were calculated from 3-D coordinates of tracked cells using Matlab [61]. T cells attached to HEVs were not included in the analysis. Arrest coefficient, motility coefficient, and meandering index were calculated as summarized in S1 Table.

### Statistical Analysis

One-way ANOVA or a Kruskal-Wallis test was used for all multiple group comparisons. Post-tests are indicated in figure legends. Numerical data and statistical analyses of all figures are available in S1 Data. Differences with a *p*-value < 0.05 were considered statistically significant. GraphPad Prism 5 was used for all statistical analyses.

## Supporting Information

S1 Data Numerical data and statistical tests of all figures. (XLS)

S1 Table Description of the morphological, topological and migration parameters. * Dimensionless unit. ** Integer number.
(DOCX)

S1 Fig FRC network restoration kinetics following complete ablation. (A) Two IP injections of 8 ng DT per gram mouse weight were given to *Ccl19*^*eyfp/idtr*^ at the indicated time points, and the analysis was performed on day 0 (complete ablation), day 14 (partial restoration), and day 28 (complete restoration). (B) Confocal microscopy Z-stack images of the T cell zone with approximate size 304 x 304 x 30 μm stained with EYFP and PDPN. (C) Global 3-D reconstruction of the EYFP^+^ FRC network. Data are representative of 3–5 mice per group. Scale bars represent 30 μm.
(TIF)

S2 Fig Impact of DT-graded ablation of the FRC network on LN architecture. (A) Representative 2-D overview images of whole LN sections of *Ccl19*^*eyfp/idtr*^ mice injected twice IP with indicated doses of DT stained against the indicated markers from 2–5 mice per group. Scale bars represent 300 μm. The star indicates a partially ablated FRC network in one LN lobe.
(TIF)

S3 Fig Impact of DT-graded FRC ablation on LN cellularity and intranodal migration. Flow cytometric analysis of total numbers of CD4^+^ T cells (A) and B220^+^ B cells (B) in LNs of *Ccl19*^*idtr*^ mice injected twice IP with indicated doses of DT. (C) Two-photon microscopy analysis of meandering index of adoptively transferred CD8^+^ T cells into *Ccl19*^*idtr*^ mice injected twice IP with indicated doses of DT. (D) Three-dimensional Z-stack images of the T cell zone FRC network of PBS-treated *Ccl19*^*eyfp/idtr*^ control mice (0 ng/g DT) against indicated markers. Confocal microscopy analysis of adoptively transferred TCR-transgenic CD8^+^ T cells (Spiky) in LNs performed on day 2 post immunization with DC-targeting viral particles. (E) Zoom-in panels of the area indicated by rectangle in (D). Scale bars represent 30 μm (D) and 10 μm (E). Data represent mean ± standard error of the mean (SEM) for 6–20 mice per group from three independent experiments (A–B). Data represent mean ± standard deviation (SD) for 5–10 datasets from 2–3 mice per group from two independent experiments (C). * *p* < 0.05, ** *p* < 0.01, *** *p* < 0.001 (one-way ANOVA with Tukey’s post-test [A–B] or Kruskal-Wallis test with Dunn’s post-test [C]). ns, not significant.
(TIF)

S4 Fig Global multiparameter correlational analysis. (A) Heat map of Pearson correlation coefficients between the following parameters in four readouts: (1) functional biology—number of FRCs in the T cell zone determined by microscopy, total numbers of CD45^+^, CD4^+^, CD8^+^, B220^+^ cells, and CD11c^+^ DCs per LN by flow cytometry, total number of Thy1.1^+^CD8^+^ T cells per LN, and relative percentage of Thy1.1^+^CFSE^low^ proliferating T cells; (2) cell migration—average cell speed, motility coefficient (MC), and arrest coefficient (AC); (3) single-cell morphology—cell surface area (A), cell volume (V), minimal distances between FRCs (Dist), sphericity (S), and number of connected protrusions per FRC (Nconn); and (4) network topology—total number of nodes (N) and edges (E), average number of edges per node (avg E), average clustering coefficient (C), network robustness (R), and small-world parameters sigma and omega. Colors indicate positive correlation (red), anticorrelation (blue), or no correlation (white). Values in the main diagonal were omitted for visualization purposes. Data represent linear regression models using Pearson correlation for mean values ± SD of the indicated parameters for each DT dose 0, 0.5, 1, 2, and 8 ng/g in *Ccl19*^*idtr*^ mice with number of mice indicated in the legends of Figs 4–7.
(TIF)

S1 Video FRC network 3-D reconstruction and analysis pipeline. Confocal microscopy analysis was performed on whole LN histological sections of naive adult *Ccl19*^*eyfp*^ mice stained for EYFP, PDPN, and DAPI. One to two T cell zones (approximately 300 x 300 x 30 μm) per LN were acquired in high resolution in order to generate the representative T cell zone FRC network. A small zoom-in area with several single FRCs was selected for visualization purposes. The cell body was stained by EYFP, the cell protrusions were accurately visualized by PDPN, and DAPI staining was used to identify cell nuclei. In order to identify single FRCs, the 3-D reconstructions of EYFP^+^ FRCs (white) were masked to the DAPI channel. The whole EYFP^+^ network was then 3-D reconstructed using an automatic threshold, and the surface area and volume of the whole FRC network was calculated. FRCs suitable for single-cell analysis (yellow) were selected and their morphological parameters were determined (e.g., single cell surface area, volume, and sphericity). Centers of homogeneous mass of FRCs were determined based on the 3-D reconstructions and selected as nodes for topological analysis. The FRC network edges (connections) were traced based on the physical connections between neighboring FRCs, and an undirected, unweighted network graph was generated. The adjacency matrix of the FRC network containing connectivity information was created from the network graph and imported into RStudio for subsequent topological network analysis.
(MP4)

S2 Video FRC network fragmentation kinetics under random node removal. Topological model of the 3-D-reconstructed FRC network (0 ng/g) under random node removal for one simulation (left panel). Fraction of nodes removed *f* and number of remaining nodes / initial number of nodes are displayed in the bottom left. Green nodes denote the largest connected cluster of nodes, and blue nodes denote fragmented clusters. Perturbation analysis of the FRC network had the following network parameters: relative size of the largest connected cluster (compared to initial network size), shortest path length, local clustering coefficient, and small-world σ factor over fraction of nodes removed. Data represent mean ± SD over 1,000 simulations for one representative FRC network dataset. Dashed lines represent minimal damage line (top left), network threshold point at maximal value of shortest path length (vertical line, top right), local clustering coefficient of the initial FRC network (horizontal line, bottom left), and the small-world σ factor of the initial FRC network (horizontal line, bottom right).
(MP4)

S3 Video Normal T cell movement in LN T cell zone. Intravital two-photon microscopy analysis of T cell movement in LN T cell zone from PBS-treated control mice. Three-dimensional reconstructions of T cells (cyan) and high endothelial venules (grey) in a representative T cell zone 300 x 300 x 64 μm. T cell tracking displayed with time color coding indicating imaging time in 20 s intervals up to 30 min. Scale bar represents 30 μm.
(MP4)

S4 Video Impaired T cell movement in LN T cell zone. Intravital two-photon microscopy analysis of T cell movement in LN T cell zone from 8 ng/g DT-treated mice. Three-dimensional reconstructions of T cells (cyan) and high endothelial venules (grey) in a representative T cell zone 200 x 200 x 64 μm. T cell tracking displayed with time color coding indicating imaging time in 20 s intervals up to 30 min. Scale bar represents 20 μm.
(MP4)

## Abbreviations

7AAD: 7-amino-actinomycin D
CCL19: C-C motif chemokine 19
CLEC2: C-type lectin-like receptor 2
CXCL12: C-X-C motif chemokine 12
DAPI: 4′,6-diamidin-2-phenylindol
DC: dendritic cell
DT: diphtheria toxin
DTR: diphtheria toxin receptor
EYFP: enhanced yellow fluorescent protein
FRC: fibroblastic reticular cell
IL-7: interleukin 7
IP: intraperitoneal(ly)
IQR: interquartile range
IV: intravenous(ly)
LN: lymph node
PBS: phosphate-buffered saline
PDPN: podoplanin
SD: standard deviation
SEM: standard error of the mean
SLO: secondary lymphoid organ

## References

[1] Junt T, Scandella E, Ludewig B. Form follows function: lymphoid tissue microarchitecture in antimicrobial immune defence. Nat Rev Immunol. 2008;8: 764–775. 10.1038/nri2414 18825130

[2] Blattman JN, Antia R, Sourdive DJ, Wang X, Kaech SM, Murali-Krishna K, et al Estimating the precursor frequency of naive antigen-specific CD8 T cells. J Exp Med. 2002;195: 657–664. 11877489 10.1084/jem.20001021 PMC2193761

[3] Moon JJ, Chu HH, Pepper M, McSorley SJ, Jameson SC, Kedl RM, et al Naive CD4(+) T cell frequency varies for different epitopes and predicts repertoire diversity and response magnitude. Immunity. 2007;27: 203–213. 17707129 10.1016/j.immuni.2007.07.007 PMC2200089

[4] Scandella E, Bolinger B, Lattmann E, Miller S, Favre S, Littman DR, et al Restoration of lymphoid organ integrity through the interaction of lymphoid tissue-inducer cells with stroma of the T cell zone. Nat Immunol. 2008;9: 667–675. 10.1038/ni.1605 18425132

[5] Chai Q, Onder L, Scandella E, Gil-Cruz C, Perez-Shibayama C, Cupovic J, et al Maturation of Lymph Node Fibroblastic Reticular Cells from Myofibroblastic Precursors Is Critical for Antiviral Immunity. Immunity. 2013;38: 1013–1024. 10.1016/j.immuni.2013.03.012 23623380 PMC7111182

[6] Link A, Vogt TK, Favre S, Britschgi MR, Acha-Orbea H, Hinz B, et al Fibroblastic reticular cells in lymph nodes regulate the homeostasis of naive T cells. Nat Immunol. 2007;8: 1255–1265. 17893676 10.1038/ni1513

[7] Cremasco V, Woodruff MC, Onder L, Cupovic J, Nieves-Bonilla JM, Schildberg FA, et al B cell homeostasis and follicle confines are governed by fibroblastic reticular cells. Nat Immunol. 2014;15: 973–981. 10.1038/ni.2965 25151489 PMC4205585

[8] Mionnet C, Mondor I, Jorquera A, Loosveld M, Maurizio J, Arcangeli ML, et al Identification of a new stromal cell type involved in the regulation of inflamed B cell follicles. PLoS Biol. 2013;11: e1001672 10.1371/journal.pbio.1001672 24130458 PMC3794863

[9] Rodda LB, Bannard O, Ludewig B, Nagasawa T, Cyster JG. Phenotypic and Morphological Properties of Germinal Center Dark Zone Cxcl12-Expressing Reticular Cells. J Immunol. 2015;195: 4781–4791. 10.4049/jimmunol.1501191 26453751 PMC4637241

[10] Fletcher AL, Acton SE, Knoblich K. Lymph node fibroblastic reticular cells in health and disease. Nat Rev Immunol. 2015;15: 350–361. 25998961 10.1038/nri3846 PMC5152733

[11] Brown FD, Turley SJ. Fibroblastic Reticular Cells: Organization and Regulation of the T Lymphocyte Life Cycle. J Immunol. 2015;194: 1389–1394. 10.4049/jimmunol.1402520 25663676 PMC4324549

[12] Newman ME. The structure and function of complex networks. SIAM Review. 2003;45: 167–256.

[13] Barabasi AL, Oltvai ZN. Network biology: understanding the cell's functional organization. Nat Rev Genet. 2004;5: 101–113. 14735121 10.1038/nrg1272

[14] Albert R, Jeong H, Barabasi AL. Error and attack tolerance of complex networks. Nature. 2000;406: 378–382. 10935628 10.1038/35019019

[15] Cohen R, Erez K, ben-Avraham D, Havlin S. Resilience of the internet to random breakdowns. Phys Rev Lett. 2000;85: 4626–4628. 11082612 10.1103/PhysRevLett.85.4626

[16] Newman ME. Coauthorship networks and patterns of scientific collaboration. Proc Natl Acad Sci U S A. 2004;101 Suppl 1: 5200–5205. 14745042 10.1073/pnas.0307545100 PMC387296

[17] Albert R, Albert I, Nakarado GL. Structural vulnerability of the North American power grid. Phys Rev E Stat Nonlin Soft Matter Phys. 2004;69: 025103 14995510 10.1103/PhysRevE.69.025103

[18] Guimera R, Mossa S, Turtschi A, Amaral LA. The worldwide air transportation network: Anomalous centrality, community structure, and cities' global roles. Proc Natl Acad Sci U S A. 2005;102: 7794–7799. 15911778 10.1073/pnas.0407994102 PMC1142352

[19] Jeong H, Tombor B, Albert R, Oltvai ZN, Barabasi AL. The large-scale organization of metabolic networks. Nature. 2000;407: 651–654. 11034217 10.1038/35036627

[20] Ideker T, Thorsson V, Ranish JA, Christmas R, Buhler J, Eng JK, et al Integrated genomic and proteomic analyses of a systematically perturbed metabolic network. Science. 2001;292: 929–934. 11340206 10.1126/science.292.5518.929

[21] Rual JF, Venkatesan K, Hao T, Hirozane-Kishikawa T, Dricot A, Li N, et al Towards a proteome-scale map of the human protein-protein interaction network. Nature. 2005;437: 1173–1178. 16189514 10.1038/nature04209

[22] Bassett DS, Bullmore E. Small-world brain networks. Neuroscientist. 2006;12: 512–523. 17079517 10.1177/1073858406293182

[23] Achard S, Salvador R, Whitcher B, Suckling J, Bullmore E. A resilient, low-frequency, small-world human brain functional network with highly connected association cortical hubs. J Neurosci. 2006;26: 63–72. 16399673 10.1523/JNEUROSCI.3874-05.2006 PMC6674299

[24] Erdos P, Renyi A. On the evoluation of random graphs. Publ Math Inst Hung Acad Sci. 1960;5: 17–61.

[25] Barabasi AL, Albert R. Emergence of scaling in random networks. Science. 1999;286: 509–512. 10521342 10.1126/science.286.5439.509

[26] Cohen R, Havlin S. Scale-free networks are ultrasmall. Phys Rev Lett. 2003;90: 058701 12633404 10.1103/PhysRevLett.90.058701

[27] Watts DJ, Strogatz SH. Collective dynamics of 'small-world' networks. Nature. 1998;393: 440–442. 9623998 10.1038/30918

[28] Reijneveld JC, Ponten SC, Berendse HW, Stam CJ. The application of graph theoretical analysis to complex networks in the brain. Clin Neurophysiol. 2007;118: 2317–2331. 17900977 10.1016/j.clinph.2007.08.010

[29] Kitano H. Systems biology: a brief overview. Science. 2002;295: 1662–1664. 11872829 10.1126/science.1069492

[30] Kaye PM, Svensson M, Ato M, Maroof A, Polley R, Stager S, et al The immunopathology of experimental visceral leishmaniasis. Immunol Rev. 2004;201:239–53.: 239–253. 15361245 10.1111/j.0105-2896.2004.00188.x

[31] Bajenoff M, Egen JG, Koo LY, Laugier JP, Brau F, Glaichenhaus N, et al Stromal cell networks regulate lymphocyte entry, migration, and territoriality in lymph nodes. Immunity. 2006;25: 989–1001. 17112751 10.1016/j.immuni.2006.10.011 PMC2692293

[32] Onder L, Narang P, Scandella E, Chai Q, Iolyeva M, Hoorweg K, et al IL-7-producing stromal cells are critical for lymph node remodeling. Blood. 2012;120: 4675–4683. 10.1182/blood-2012-03-416859 22955921 PMC3952724

[33] Humphries MD, Gurney K, Prescott TJ. The brainstem reticular formation is a small-world, not scale-free, network. Proc Biol Sci. 2006;273: 503–511. 16615219 10.1098/rspb.2005.3354 PMC1560205

[34] Humphries MD, Gurney K. Network 'small-world-ness': a quantitative method for determining canonical network equivalence. PLoS ONE. 2008;3: e0002051 10.1371/journal.pone.0002051 18446219 PMC2323569

[35] Telesford QK, Joyce KE, Hayasaka S, Burdette JH, Laurienti PJ. The ubiquity of small-world networks. Brain Connect. 2011;1: 367–375. 10.1089/brain.2011.0038 22432451 PMC3604768

[36] Strogatz SH. Exploring complex networks. Nature. 2001;410: 268–276. 11258382 10.1038/35065725

[37] Astarita JL, Cremasco V, Fu J, Darnell MC, Peck JR, Nieves-Bonilla JM, et al The CLEC-2-podoplanin axis controls the contractility of fibroblastic reticular cells and lymph node microarchitecture. Nat Immunol. 2015;16: 75–84. 10.1038/ni.3035 25347465 PMC4270928

[38] Braun A, Worbs T, Moschovakis GL, Halle S, Hoffmann K, Bolter J, et al Afferent lymph-derived T cells and DCs use different chemokine receptor CCR7-dependent routes for entry into the lymph node and intranodal migration. Nat Immunol. 2011;12: 879–887. 10.1038/ni.2085 21841786

[39] Cupovic J, Onder L, Gil-Cruz C, Weiler E, Caviezel-Firner S, Perez-Shibayama C, et al Central Nervous System Stromal Cells Control Local CD8(+) T Cell Responses during Virus-Induced Neuroinflammation. Immunity. 2016;44: 622–633. 10.1016/j.immuni.2015.12.022 26921107 PMC7111064

[40] Cervantes-Barragan L, Zust R, Maier R, Sierro S, Janda J, Levy F, et al Dendritic cell-specific antigen delivery by coronavirus vaccine vectors induces long-lasting protective antiviral and antitumor immunity. MBio. 2010;1: e00171–10. 10.1128/mBio.00171-10 20844609 PMC2939679

[41] Perez-Shibayama C, Gil-Cruz C, Nussbacher M, Allgauer E, Cervantes-Barragan L, Zust R, et al Dendritic cell-specific delivery of Flt3L by coronavirus vectors secures induction of therapeutic antitumor immunity. PLoS ONE. 2013;8: e81442 10.1371/journal.pone.0081442 24312302 PMC3842931

[42] Krummel MF, Bartumeus F, Gerard A. T cell migration, search strategies and mechanisms. Nat Rev Immunol. 2016;16: 193–201. 10.1038/nri.2015.16 26852928 PMC4869523

[43] Acton SE, Astarita JL, Malhotra D, Lukacs-Kornek V, Franz B, Hess PR, et al Podoplanin-rich stromal networks induce dendritic cell motility via activation of the C-type lectin receptor CLEC-2. Immunity. 2012;37: 276–289. 10.1016/j.immuni.2012.05.022 22884313 PMC3556784

[44] Bullmore E, Sporns O. Complex brain networks: graph theoretical analysis of structural and functional systems. Nat Rev Neurosci. 2009;10: 186–198. 10.1038/nrn2575 19190637

[45] Sixt M, Kanazawa N, Selg M, Samson T, Roos G, Reinhardt DP, et al The conduit system transports soluble antigens from the afferent lymph to resident dendritic cells in the T cell area of the lymph node. Immunity. 2005;22: 19–29. 15664156 10.1016/j.immuni.2004.11.013

[46] Kelch ID, Bogle G, Sands GB, Phillips AR, LeGrice IJ, Rod DP. Organ-wide 3D-imaging and topological analysis of the continuous microvascular network in a murine lymph node. Sci Rep. 2015;5: 16534 10.1038/srep16534 26567707 PMC4645097

[47] Mayer J, Swoger J, Ozga AJ, Stein JV, Sharpe J. Quantitative measurements in 3-dimensional datasets of mouse lymph nodes resolve organ-wide functional dependencies. Comput Math Methods Med. 2012;28431: 128431. 10.1155/2012/128431 PMC3461256 23049616

[48] Lee M, Mandl JN, Germain RN, Yates AJ. The race for the prize: T-cell trafficking strategies for optimal surveillance. Blood. 2012;120: 1432–1438. 10.1182/blood-2012-04-424655 22773385 PMC3423781

[49] Grossman Z, Min B, Meier-Schellersheim M, Paul WE. Concomitant regulation of T-cell activation and homeostasis. Nat Rev Immunol. 2004;4: 7–15. 10.1038/nri1355 15122204

[50] Ludewig B, Stein JV, Sharpe J, Cervantes-Barragan L, Thiel V, Bocharov G. A global "imaging'' view on systems approaches in immunology. Eur J Immunol. 2012;42: 3116–3125. 10.1002/eji.201242508 23255008

[51] Bogle G, Dunbar PR. Simulating T-cell motility in the lymph node paracortex with a packed lattice geometry. Immunol Cell Biol. 2008;86: 676–687. 10.1038/icb.2008.60 18711399 PMC2713783

[52] Bogle G, Dunbar PR. Agent-based simulation of T-cell activation and proliferation within a lymph node. Immunol Cell Biol. 2010;88: 172–179. 10.1038/icb.2009.78 19884904

[53] Baldazzi V, Paci P, Bernaschi M, Castiglione F. Modeling lymphocyte homing and encounters in lymph nodes. BMC Bioinformatics. 2009;10: 387 10.1186/1471-2105-10-387 19939270 PMC2790470

[54] Tang J, van PN, Kastenmuller W, Germain RN. The future of immunoimaging—deeper, bigger, more precise, and definitively more colorful. Eur J Immunol. 2013;43: 1407–1412. 10.1002/eji.201243119 23568494 PMC3748132

[55] Kumar V, Scandella E, Danuser R, Onder L, Nitschke M, Fukui Y, et al Global lymphoid tissue remodeling during a viral infection is orchestrated by a B cell-lymphotoxin-dependent pathway. Blood. 2010;115: 4725–4733. 10.1182/blood-2009-10-250118 20185585

[56] Oh SW, Harris JA, Ng L, Winslow B, Cain N, Mihalas S, et al A mesoscale connectome of the mouse brain. Nature. 2014;508: 207–214. 10.1038/nature13186 24695228 PMC5102064

[57] Buch T, Heppner FL, Tertilt C, Heinen TJ, Kremer M, Wunderlich FT, et al A Cre-inducible diphtheria toxin receptor mediates cell lineage ablation after toxin administration. Nat Methods. 2005;2: 419–426. 15908920 10.1038/nmeth762

[58] Schneider CM, Moreira AA, Andrade JS, Jr., Havlin S, Herrmann HJ. Mitigation of malicious attacks on networks. Proc Natl Acad Sci U S A. 2011;108: 3838–3841. 10.1073/pnas.1009440108 21368159 PMC3053993

[59] Kyburz D, Aichele P, Speiser DE, Hengartner H, Zinkernagel RM, Pircher H. T cell immunity after a viral infection versus T cell tolerance induced by soluble viral peptides. Eur J Immunol. 1993;23: 1956–1962. 8344359 10.1002/eji.1830230834

[60] Soriano SF, Hons M, Schumann K, Kumar V, Dennier TJ, Lyck R, et al In vivo analysis of uropod function during physiological T cell trafficking. J Immunol. 2011;187: 2356–2364. 10.4049/jimmunol.1100935 21795598

[61] Mempel TR, Henrickson SE, von Andrian UH. T-cell priming by dendritic cells in lymph nodes occurs in three distinct phases. Nature. 2004;427: 154–159. 14712275 10.1038/nature02238

