# Supplementary material for: Topological Small-World Organization of the Fibroblastic Reticular Cell Network Determines Lymph Node Functionality
Source: PLoS Biol. 2016 Jul 14;14(7):e1002515. doi: 10.1371/journal.pbio.1002515 (PMC4945005; doi:10.1371/journal.pbio.1002515)
Supplement: S1 Table — * Dimensionless unit. ** Integer number. (DOCX) [file pbio.1002515.s002.docx]

| Parameter | Unit | Description | Ref |
| --- | --- | --- | --- |
| Surface area | μm^2^ | Total area of the surface of a solid 3D object. |  |
| Volume | μm^3^ | Space that is occupied by a solid 3D object with an enclosed surface. |  |
| Sphericity  (*ψ*) | % | Morphometric characteristic of shape which represents how spherical an object is. Sphericity *ψ* is defined as the ratio of the surface area of a sphere with the same volume *V* as the object to the surface area of the object *A*:  $\psi=\frac{\pi^{\frac{1}{3}} \left( 6V \right)^{\frac{2}{3}}}{A} , 0<\psi\leq1$  In the case of a sphere with volume $V=\frac{4}{3}{\pi r}^{3}$ and surface area $A=4{\pi r}^{2}$, the sphericity formula is reduced to $\psi=1$. For 3D objects which have $A\gg V$, the sphericity $\psi\to0$. | [1] |
| Compactness  (*C*) | dim^*^ | Measure of shape compactness which represents the degree to which a shape is compact. The classical compactness of a solid 3D object is defined as the given ratio between surface area *A* and volume *V* of the object:  $C=\frac{A^{3}}{V^{2}} , 36\pi\leq C<\infty$  This measure is dimensionless and is minimized by a sphere with volume $V=\frac{4}{3}{\pi r}^{3}$ and surface area $A=4{\pi r}^{2}$, having the most compact shape for $C=36\pi$. | [2] |
| Minimal distance  ${(d}_{min})$ | μm | Distance between centers of mass of FRCs in 3D Euclidean space, which is then minimized ($d_{min}$) by finding the nearest FRC neighbor. The distance between two FRCs *i* and *j* with Cartesian coordinates $\left( x_{i},y_{i},z_{i} \right)$ and $\left( x_{j},y_{j},z_{j} \right)$ respectively, is given by:  $d\left( i,j \right)=\sqrt{\left( x_{j}-x_{i} \right)^{2}+\left( y_{j}-y_{i} \right)^{2}+\left( z_{j}-z_{i} \right)^{2}}$ |  |
| Connected protrusions | # ^**^ | Number of protrusions per 3D reconstructed FRC body, counted before the first branching point and connected to another FRC. |  |
| Number of nodes | # ^**^ | Total number of nodes *n* connected in a network. |  |
| Number of edges | # ^**^ | Total number of edges for all nodes in a network. |  |
| Average number of edges per FRC  $(\bar{e})$ | # ^**^ | The arithmetic mean of the number of edges $e_{i}$ per node *i*, for the network with *n* nodes:  $\bar{e}=\frac{1}{n}\sum_{i=1}^{n} e_{i}$ |  |
| Average local clustering coefficient  $(\bar{C})$ | % | The local clustering coefficient $c_{i}$ of a node *i* is defined as the number of edges $e_{i}$ among neighbors of *i* divided by the total possible number of edges among its neighbors:  $c_{i}=\frac{2}{\delta_{i}\left( \delta_{i}-1 \right)}e_{i} , 0\leq c_{i}\leq1$  where $\delta_{i}$ represents the number of neighbors of node *i*.  The average local clustering coefficient of a network with *n* nodes is the arithmetic mean of clustering coefficients of all the nodes:  $\bar{C}=\frac{1}{n}\sum_{{i=1; \delta}_{i}>1}^{n} c_{i} , 0\leq\bar{C}\leq1$ | [3] |
| Average shortest path length  ($\bar{L}$) | dim^*^ | The average shortest path length *L* of a network is determined as the arithmetic mean of all pairs of shortest distances between nodes *i* and *j*:  $\bar{L}=\frac{2}{n\left( n-1 \right)}\sum_{i=1}^{n} \sum_{j=i+1}^{n} l_{ij} , 1\leq\bar{L}<\infty$ | [4] |
|  |  | where $l_{ij}$ is the length (number of edges) of the shortest path between nodes *i* and *j*, namely how many nodes one needs to pass in order to get from node *i* to node *j*. The maximum distance $\bar{L}_{max}$ is called the diameter of the network.  In case of a complete network where all possible connections are present, all the node distances $l_{ij}=1$, thus the sum $\sum_{i=1}^{n} \sum_{j=i+1}^{n} l_{ij}=\frac{n\left( n-1 \right)}{2}$, which gives the minimal $\bar{L}_{min}=1$. |  |
| Sigma factor  (*σ*) | dim^*^ | The small-world measure $\sigma$ is determined by comparing the average clustering coefficient $\bar{C}$ and average shortest path length $\bar{L}$ of the network in question to an equivalent Erdos-Renyi random network with the same number of nodes and edges:  $\sigma=\frac{\bar{C}/{C_{R}}}{\bar{L}/{L_{R}}} , 1\leq\sigma<\infty$  where $C_{R}$ and $L_{R}$ are the average clustering coefficient and average shortest path length of the random network, respectively, averaged across 100 simulation runs of an equivalent random network.  In the case of a random network $\bar{C}=C_{R}, \bar{L}=L_{R}$, the sigma factor $\sigma=1$.  A network will be classified as a small-world network if $\bar{C}\gg C_{R}, \bar{L}\geq L_{R}$ which implies $\bar{C}/{C_{R}}\gg1$ and $\bar{L}/{L_{R}}\geq1$ and therefore $\sigma>1$. | [5,6] |
| Omega factor  (*ω*) | dim^*^ | The small-world measure $\omega$ is determined by comparing the average clustering coefficient of the network in question $\bar{C}$ to that of an equivalent lattice network $C_{L}$ and comparing the average shortest path length $\bar{L}$ to that of an equivalent Erdos-Renyi random network $L_{R}$ as follows:  $\omega=\frac{L_{R}}{\bar{L}}-\frac{\bar{C}}{C_{L}} , -1<\omega<1$  In the case of a random network $\bar{C}\ll C_{L}, {\bar{L}\approx L}_{R}$, the omega factor $\omega\to1$ for $n\to\infty$, while in the case of a lattice network $\bar{C}\approx C_{L}$, $\bar{L}\gg L_{R}$ will give rise to $\omega\to-1$.  A network will be classified as a small-world network if it has average shortest path length like a random network ${\bar{L}\approx L}_{R}$ and average clustering coefficient like a lattice network $\bar{C}\approx C_{L}$, which gives near-zero values $\omega\approx0$.  Note that it is suggested that the small-world regime spans in the following range of *ω* values: $-0.5\leq\omega\leq0.5$. Although there is no precise cut-off, the proximity to zero indicates small-world network attributes. | [7] |
| Network robustness  (*R*) | dim^*^ | Network robustness can be assessed by sequentially removing *q* nodes from a network and is defined as:  $R=\frac{1}{n}\sum_{q=1}^{n} s\left( q \right) , 0<R<0.5$  where $s\left( q \right)=m/n$ is the fraction of nodes *m* in the largest connected cluster (subgraph) when *q* nodes are sequentially removed over the number of nodes *n* of the initial network.  The largest connected subgraph must also satisfy the following two conditions:  1) Must have the largest number of connected nodes *m* and consequently the largest fraction $s\left( q \right)$.  2) The nodes *k* in the largest connected subgraph must be on average connected to at least two other nodes:  $\frac{\bar{e^{2}}}{\bar{e}}\geq2 , \bar{e^{2}}=\frac{1}{m}\sum_{k=1}^{m} e_{k}^{2} , \bar{e}=\frac{1}{m}\sum_{k=1}^{m} e_{k}$  The largest connected subgraph is selected when both conditions are maximized $max\left( s\left( q \right) \right)$ and $max\left( {\bar{e^{2}}}/\bar{e} \right)$.  If no subgraph meets these conditions, the fraction $s\left( q \right)=0$.  Robustness of a network is calculated in the range of maximal vulnerability (*R=0*) and maximal robustness (*R=0.5*). | [8,9] |
| Average speed  ($\bar{v}$) | μm/min | Average 3D speed of a cell is calculated as the mean of all cell velocities estimated at time points numbered with *t* and spanning 30 min time interval with time step $\Delta t$ as follows:  $\bar{v}=\frac{1}{T}\sum_{t=1}^{T} v\left( t \right)$  $v\left( t \right)=\frac{\Delta s}{\Delta t}=\frac{\sqrt{\left( \Delta x\left( t-1,t \right) \right)^{2}+\left( \Delta y\left( t-1,t \right) \right)^{2}+\left( \Delta z\left( t-1,t \right) \right)^{2}}}{\Delta t}$  where *T* is the number of time points for 30 min total imaging time, $v\left( t \right)$ is the estimated absolute speed of the cell over 20 sec time intervals $\Delta t$, and $\Delta x,\Delta y,\Delta z$ specify the change in 3D cell position between consecutive time points *(t-1,t)*. |  |
| Arrest coefficient | % | Percentage of the time a cell spends travelling at speed less than 4 μm/min: | [10] |
| (*AC*) |  | $AC=\frac{t\left( v_{t}<4\mu m/min \right)}{T\left( v_{t} \right)} , 0\leq AC\leq1$ |  |
| Motility coefficient  (*MC*) | μm^2^/min | The motility coefficient of a data set can be derived from the mean displacement $\Delta s$ of all cells within the data set at time point *t = 1 min*:  $MC=\frac{\pi{\cdot\Delta s}^{2}}{16t}$  The motility coefficient serves as a measure for area scanned by cells. | [11] |
| Meandering index  (*MI*) | dim^*^ | The meandering index, i.e. cell track straightness, is calculated as the ratio of cell displacement $\Delta s$ and total track length *L*:  $MI=\frac{\Delta s}{L} , 0\leq MI\leq1$  The meandering index is a measure of movement straightness. A cell moving in an exact straight line will have *MI = 1*. | [12] |

**S1 Table References:**

[1] Wadell H (1935) Volume, Shape and Roundness of Quartz Particles. J. Geol 43: 250-280.

[2] Bribiesca E (2000) A measure of compactness for 3D shapes. Comput Math Appl 40: 1275-1284.

[3] Watts DJ and Strogatz S (1998) Collective dynamics of 'small-world' networks. Nature 393: 440-442.

[4] Wiener H (1947) Structural determination of paraffin boiling points. J Am Chem Soc 69: 17-20.

[5] Humphries MD, Gurney K, Prescott TJ (2006) The brainstem reticular formation is a small-world, not scale-free, network. Proc Biol Sci 273: 503-511.

[6] Humphries MD, Gurney K (2008) Network 'small-world-ness': a quantitative method for determining canonical network equivalence. PLoS ONE 3: e0002051.

[7] Telesford QK, Joyce KE, Hayasaka S, Burdette JH, Laurienti PJ (2011) The ubiquity of small-world networks. Brain Connect 1: 367-375.

[8] Schneider CM, Moreira AA, Andrade JS Jr, Havlin S, Herrmann HJ (2011) Mitigation of malicious attacks on networks. Proc Natl Acad Sci USA. 108: 3838-3841.

[9] Cohen R, Erez K, ben-Avraham D, Havlin S (2000) Resilience of the internet to random breakdowns. Phys Rev Lett. 85: 4626-4628.

[10] Hugues S, Fetler L, Bonifaz L, Helft J, Amblard F, Amigorena S (2004) Distinct T cell dynamics in lymph nodes during the induction of tolerance and immunity. Nat Immunol. 5: 1235-1242.

[11] Beltman JB, Marée AF, de Boer RJ (2009) Analysing immune cell migration. Nat Rev Immunol. 9: 789-798.

[12] Worbs T, Mempel TR, Bölter J, von Andrian UH, Förster R (2007) CCR7 ligands stimulate the intranodal motility of T lymphocytes in vivo. J Exp Med. 204: 489-495.
