# Supplementary figures and images for: Topological Small-World Organization of the Fibroblastic Reticular Cell Network Determines Lymph Node Functionality
Source: PLoS Biol. 2016 Jul 14;14(7):e1002515. doi: 10.1371/journal.pbio.1002515 (PMC4945005; doi:10.1371/journal.pbio.1002515)

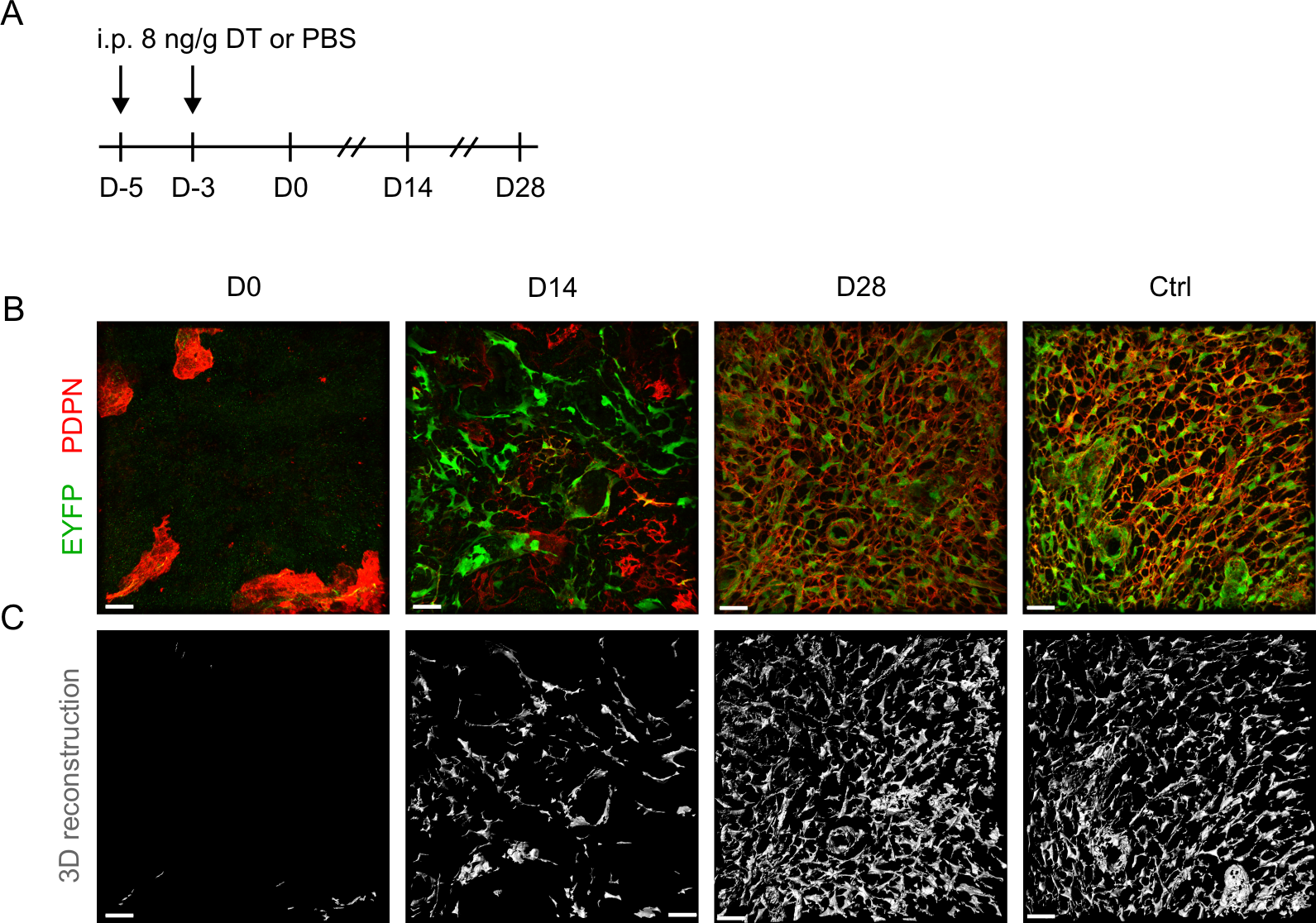

Supplement: S1 Fig — (A) Two IP injections of 8 ng DT per gram mouse weight were given to Ccl19eyfp/idtr at the indicated time points, and the analysis was performed on day 0 (complete ablation), day 14 (partial restoration), and day 28 (complete restoration). (B) Confocal microscopy Z-stack images of the T cell zone with approximate size 304 x 304 x 30 μm stained with EYFP and PDPN. (C) Global 3-D reconstruction of the EYFP+ FRC network. Data are representative of 3–5 mice per group. Scale bars represent 30 μm. (TIF) [file pbio.1002515.s003.tif]

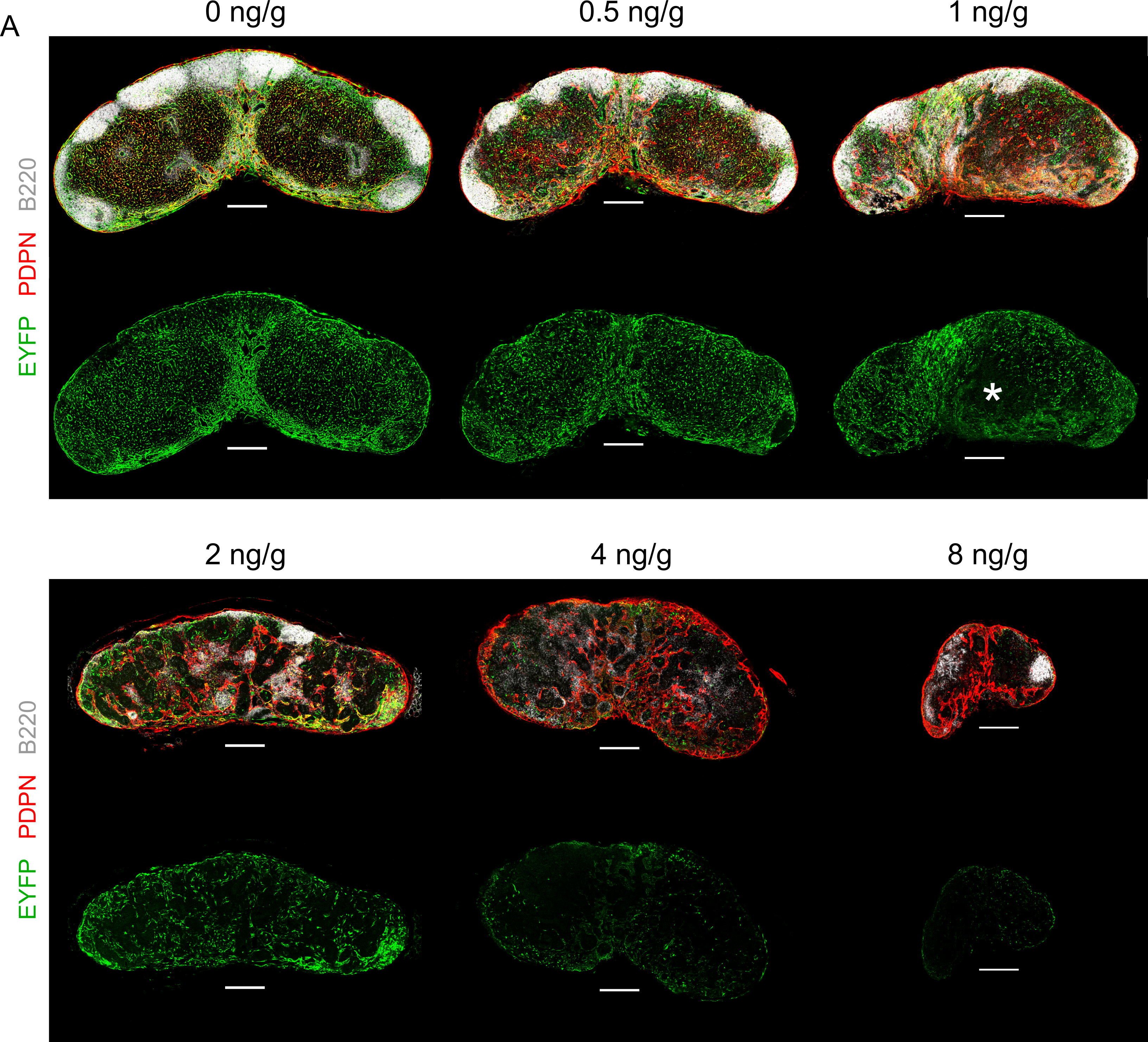

Supplement: S2 Fig — (A) Representative 2-D overview images of whole LN sections of Ccl19eyfp/idtr mice injected twice IP with indicated doses of DT stained against the indicated markers from 2–5 mice per group. Scale bars represent 300 μm. The star indicates a partially ablated FRC network in one LN lobe. (TIF) [file pbio.1002515.s004.tif]

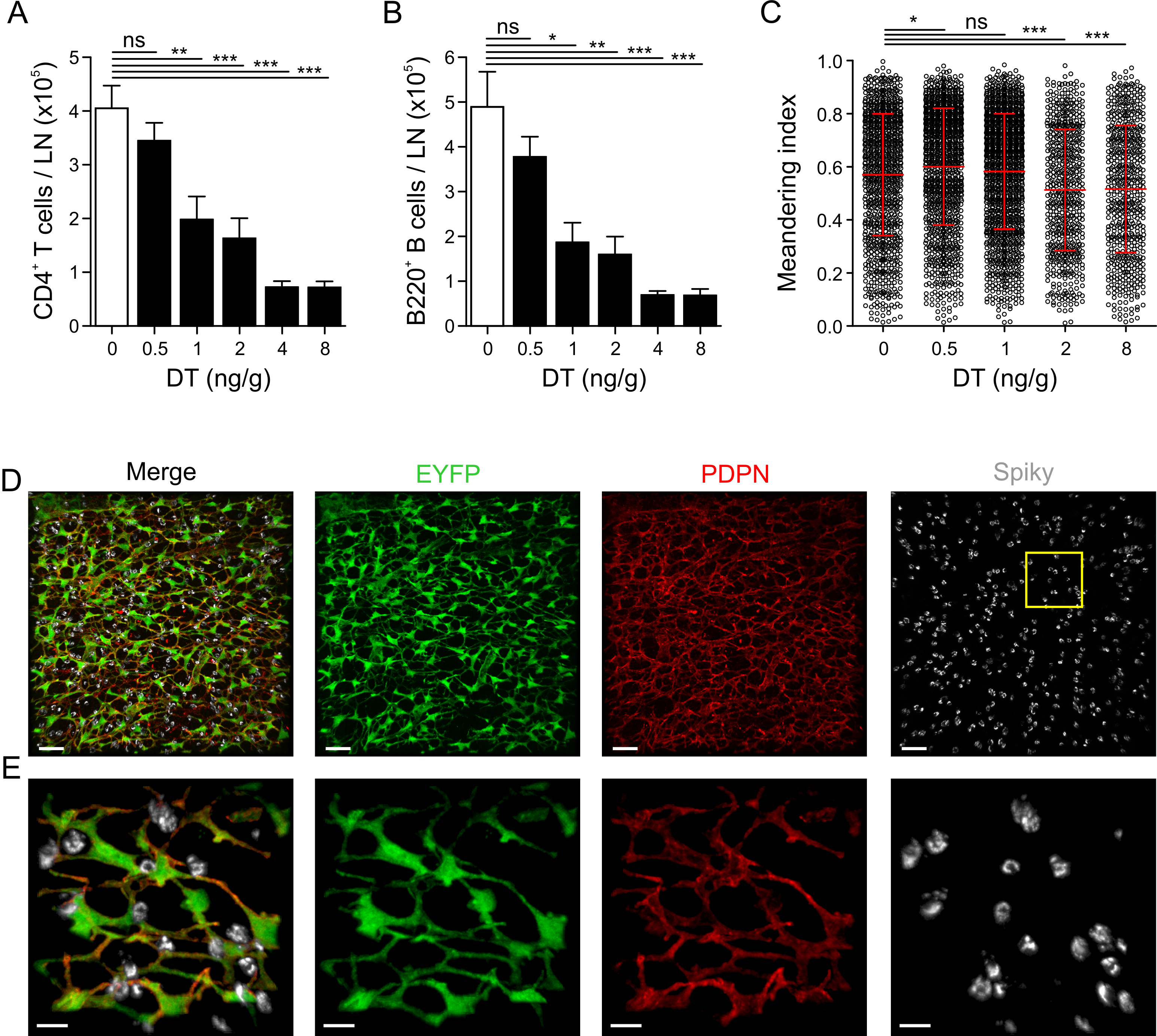

Supplement: S3 Fig — Flow cytometric analysis of total numbers of CD4+ T cells (A) and B220+ B cells (B) in LNs of Ccl19idtr mice injected twice IP with indicated doses of DT. (C) Two-photon microscopy analysis of meandering index of adoptively transferred CD8+ T cells into Ccl19idtr mice injected twice IP with indicated doses of DT. (D) Three-dimensional Z-stack images of the T cell zone FRC network of PBS-treated Ccl19eyfp/idtr control mice (0 ng/g DT) against indicated markers. Confocal microscopy analysis of adoptively transferred TCR-transgenic CD8+ T cells (Spiky) in LNs performed on day 2 post immunization with DC-targeting viral particles. (E) Zoom-in panels of the area indicated by rectangle in (D). Scale bars represent 30 μm (D) and 10 μm (E). Data represent mean ± standard error of the mean (SEM) for 6–20 mice per group from three independent experiments (A–B). Data represent mean ± standard deviation (SD) for 5–10 datasets from 2–3 mice per group from two independent experiments (C). * p < 0.05, ** p < 0.01, *** p < 0.001 (one-way ANOVA with Tukey’s post-test [A–B] or Kruskal-Wallis test with Dunn’s post-test [C]). ns, not significant. (TIF) [file pbio.1002515.s005.tif]

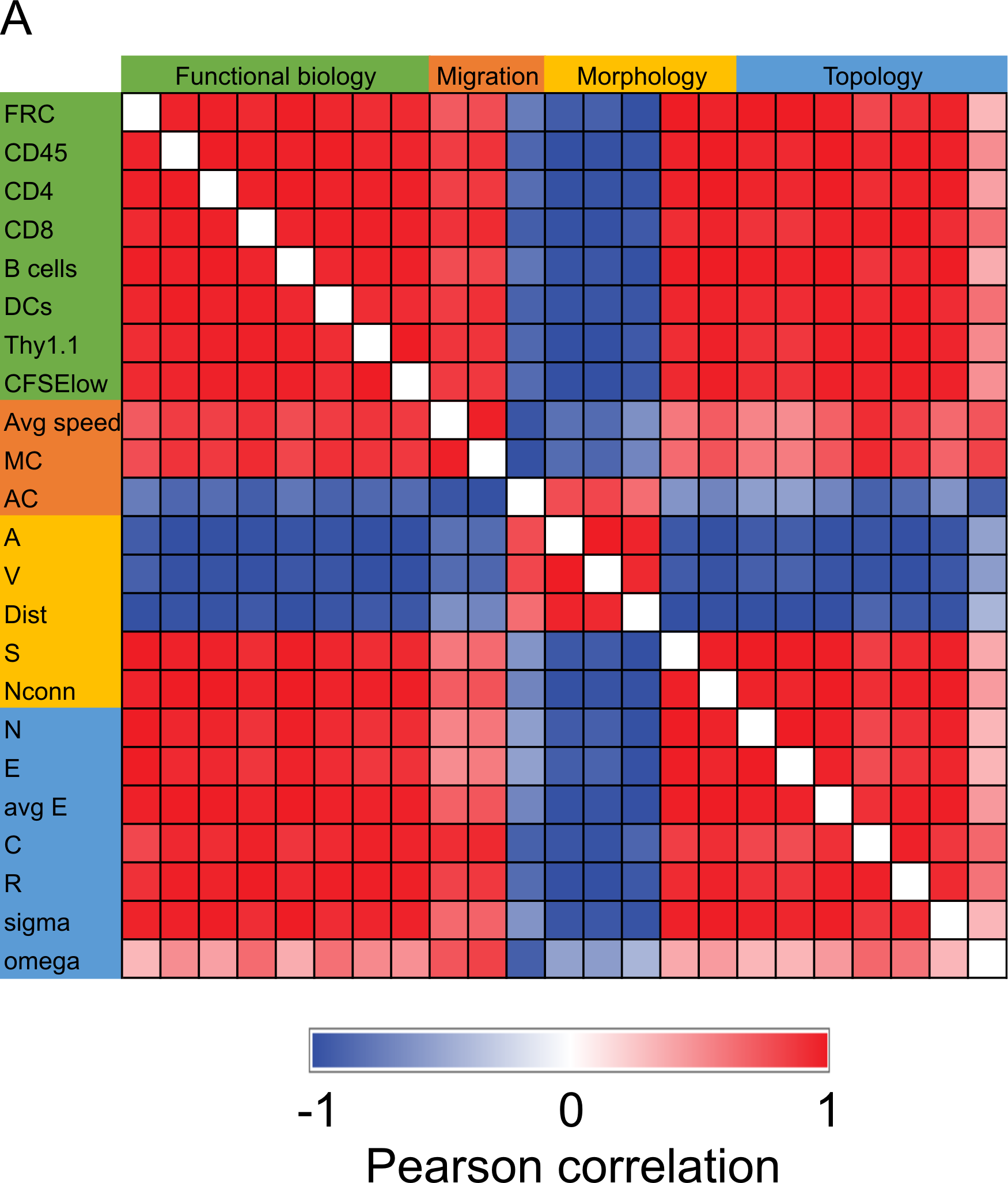

Supplement: S4 Fig — (A) Heat map of Pearson correlation coefficients between the following parameters in four readouts: (1) functional biology—number of FRCs in the T cell zone determined by microscopy, total numbers of CD45+, CD4+, CD8+, B220+ cells, and CD11c+ DCs per LN by flow cytometry, total number of Thy1.1+CD8+ T cells per LN, and relative percentage of Thy1.1+CFSElow proliferating T cells; (2) cell migration—average cell speed, motility coefficient (MC), and arrest coefficient (AC); (3) single-cell morphology—cell surface area (A), cell volume (V), minimal distances between FRCs (Dist), sphericity (S), and number of connected protrusions per FRC (Nconn); and (4) network topology—total number of nodes (N) and edges (E), average number of edges per node (avg E), average clustering coefficient (C), network robustness (R), and small-world parameters sigma and omega. Colors indicate positive correlation (red), anticorrelation (blue), or no correlation (white). Values in the main diagonal were omitted for visualization purposes. Data represent linear regression models using Pearson correlation for mean values ± SD of the indicated parameters for each DT dose 0, 0.5, 1, 2, and 8 ng/g in Ccl19idtr mice with number of mice indicated in the legends of Figs 4–7. (TIF) [file pbio.1002515.s006.tif]
